# Supplementary material for: Keep the fire burning: a survey study on the role of personal resources for work engagement and burnout in medical residents and specialists in the Netherlands
Source: BMJ Open. 2019 Nov 5;9(11):e031053. doi: 10.1136/bmjopen-2019-031053 (PMC6858141; doi:10.1136/bmjopen-2019-031053)
Supplement: Author's manuscript [file bmjopen-2019-031053.draft_revisions.pdf]

BMJ Open

BMJ Open is committed to open peer review. As part of this commitment we make the peer review history of every article we publish publicly available.

When an article is published we post the peer reviewers' comments and the authors' responses online. We also post the versions of the paper that were used during peer review. These are the versions that the peer review comments apply to.

The versions of the paper that follow are the versions that were submitted during the peer review process. They are not the versions of record or the final published versions. They should not be cited or distributed as the published version of this manuscript.

BMJ Open is an open access journal and the full, final, typeset and author-corrected version of record of the manuscript is available on our site with no access controls, subscription charges or pay-per-view fees (http://bmjopen.bmj.com).

If you have any questions on BMJ Open's open peer review process please email info.bmjopen@bmj.com

Keep the fire burning: a survey study on the role of personal resources for work engagement and burnout in medical residents and specialists

Journal:	*BMJ Open*
Manuscript ID	bmjopen-2019-031053
Article Type:	Research
Date Submitted by the Author:	15-Apr-2019

Complete List of Authors:	Solms, Lara; University of Amsterdam, Work and Organizational Psychology; Erasmus Medical Center - Sophia Children's hospital, Pediatrics<br>van Vianen, Annelies; University of Amsterdam, Work and Organizational Psychology<br>Theeboom, Tim; Vrije Universiteit Amsterdam, School of Business and Economics<br>Koen, Jessie; University of Amsterdam, Work and Organizational Psychology<br>de Pagter, Anne; Erasmus Medical Center - Sophia Children's hospital<br>De Hoog, Matthijs; Erasmus Medical Centre - Sophia Childrens Hospital, Pediatrics/ Pediatric Intensive Care Unit

Keywords:	Work engagement, Burnout, Personal resources, Professional development

SCHOLARONE™
Manuscripts

Keep the fire burning: a survey study on the role of personal resources for work engagement and burnout in medical residents and specialists

Lara Solms, MSc, Annelies E.M. van Vianen, PhD, Tim Theeboom, PhD, Jessie Koen, PhD, Anne P.J. de Pagter, MD, PhD, Matthijs de Hoog, MD, PhD

L. Solms is PhD candidate, Department of Work and Organizational Psychology, University of Amsterdam, Amsterdam, The Netherlands, PhD candidate, Department of Pediatrics, Erasmus MC-Sophia Children's Hospital, Rotterdam, The Netherlands

A.E.M van Vianen is professor of work and organizational psychology, Department of Work and Organizational Psychology, University of Amsterdam, Amsterdam, The Netherlands

T. Theeboom is assistant professor, School of Business and Economics, Vrije Universiteit Amsterdam, Amsterdam, The Netherlands

J. Koen is assistant professor, Department of Work and Organizational Psychology, University of Amsterdam, Amsterdam, The Netherlands

A.P.J. de Pagter* is a pediatrician-hematologist, Department of Pediatrics, Erasmus MC-Sophia Children's Hospital, Rotterdam, The Netherlands and LUMC, Leiden, the Netherlands

M. de Hoog* is pediatric intensivist, professor of postgraduate medical education, director of pediatric residency training & director of PICU, Erasmus MC-Sophia Children's Hospital, Rotterdam, The Netherlands

* Anne P.J. de Pagter and Matthijs de Hoog contributed equally to this paper.

Correspondence should be addressed to Anne P.J. de Pagter, Department of Pediatrics, Erasmus MC-Sophia Children's Hospital, Rotterdam, Sp-2430 Wytemaweg 80, 3015 CN Rotterdam, The Netherlands; telephone: +31107032435; e-mail: p.depagter@erasmusmc.nl

Word count: 5668

Keywords: burnout, work engagement, personal resources, professional development

For peer review only

ABSTRACT

Objectives

The high prevalence of burnout among medical residents and specialists raises concerns about the stressful demands in healthcare. This study investigated which job demands and job- and personal resources are associated with work engagement and burnout, and whether the effects of these demands and resources differ for medical residents and specialists.

Design

In a survey study among residents and specialists, we assessed job demands, job resources, personal resources, work engagement and burnout symptoms using validated questionnaires (January to December 2017). Results were analyzed using multivariate generalized linear model, ordinary least squares regression analyses, and path analyses.

Setting

Six academic and general hospitals in the Netherlands.

Participants

A total number of 124 residents and 69 specialists participated in this study. Participants worked in the fields of pediatrics, internal medicine, and neurology.

Results

The associations of job and personal resources with burnout and work engagement differed for residents and specialists. Psychological capital was associated with burnout only for specialists ($b = -.58$, $p < .001$), whereas psychological flexibility was associated with burnout only for residents ($b = -.31$, $p < .001$). Colleague support ($b = .49$, $p < .001$) and self-compassion ($b = -.33$, $p = .004$) were associated with work engagement only for specialists.

Conclusions

This study suggests that particularly personal resources safeguard the work engagement and lessen the risk of burnout of residents and specialists. Both residents and specialists benefit

For peer review only - http://bmjopen.bmj.com/site/about/guidelines.xhtml

from psychological capital to maintain optimal functioning. In addition, residents benefit from psychological flexibility while specialists benefit from colleague support. Personal resources seem important protective factors for physicians' work engagement and well-being. When promoting physician well-being, a one-size-fits-all approach might not be effective but, instead, interventions should be tailored to the specific needs of specialists and residents.

Article summary

Strengths and limitations of this study

- The participation of both residents and specialists from different specialties and the high response rate (78%) allow for a realistic display of the demands and resources present during different career stages.
- This is the first study to show that the personal resources psychological capital, psychological flexibility, and self-compassion are important protective factors for residents and/or staff when fostering work engagement and combatting burnout.
- This study reveals that different demands and resources relate to burnout and work engagement for specialists and residents, pointing to unique problems that require unique solutions.
- All data are based on self-reports, which limits drawing strong conclusions about objectively existing job characteristics and their associations with burnout and work engagement.
- Although theory and research point to causal relationships between demands and burnout and resources and work engagement, the study is limited by the fact that its cross-sectional design does not infer causality.

For peer review only

For peer review only - http://bmjopen.bmj.com/site/about/guidelines.xhtml

INTRODUCTION

Healthcare professionals are well-known for their high work engagement, that is, their absorption by their work and dedication to patient care.[1-2] Unfortunately, such vigor and dedication can also have a flipside. Healthcare professionals work in a very complex system with long and irregular working hours and need to deal with various high job demands such as time pressure, emotionally taxing patient-interactions, work-family conflict and job insecurity. These high job demands cause alarming high rates of burnout symptoms such as emotional exhaustion and cynicism in healthcare compared to other professions.[3-7]Among physicians, medical residents seem to be especially at risk for burnout, with burnout symptoms often being reported by one out of four residents.[3, 8-10] This is hardly surprising: In addition to the job demands that all physicians face, residents also face high educational demands, need to get used to clinical rotations and shifts, experience high responsibility yet limited autonomy, and experience high uncertainty about their future career.[7,11] Additionally, residency marks a period that is characterized by stressful and demanding life events such as marriage and getting children.

Obviously, burnout has a strong negative impact on healthcare professionals and their employers. Most important, burnout symptoms are associated with decreased quality of care through delayed decision making, unprofessional work behaviors (i.e., conflict), and suboptimal patient care (i.e., not adequately discussing treatment options with patients or making medical errors).[7-9] Also, burnout is accompanied by long-term sickness leave and early retirement.[7] It is the highly critical work-environment of healthcare professionals and the high-stake consequences of their functioning that call for a deeper understanding of the demands and resources that relate to the work engagement and well-being (i.e., a lack of burnout) of these professionals. Similarly, it is important to understand which factors can

For peer review only - http://bmjopen.bmj.com/site/about/guidelines.xhtml

help physicians to stay motivated in spite of the demands they face. An important result of motivation is work engagement, a positive work-related state of mind characterized by vigor, dedication, and absorption.[1,12] Work engagement has been linked to employee performance across professions,[13-14] including healthcare.[15] Healthcare professionals who are engaged are also less likely to commit medical errors.[16] Consequently, both (a lack of) burnout and work engagement are important components for the optimal functioning of healthcare professionals.

Earlier research conceptualized burnout and work engagement as opposite poles of a continuum that are mutually exclusive.[17] Recent research[18] has evidenced that burnout and work engagement are (negatively) related, yet different constructs (i.e., low burnout does not necessarily imply high engagement). Furthermore, these studies have shown that job characteristics that are associated with the prevalence of burnout are different from those that are associated with work engagement. Surprisingly, few of these studies were carried out in health care settings . Yet, knowledge of the job characteristics that relate to burnout and work engagement among healthcare professionals is essential to develop tailored interventions (e.g., training, coaching) that support these professionals in their optimal functioning.[19]

Theoretical Framework: The Job Demands – Resources Model

The Job Demands - Resources (JD-R) model[12] proposes that work engagement and well-being are promoted when (healthcare) professionals have job resources that help them to cope with high job demands and that bolster their motivation. Generally, the JD-R model differentiates between two universal types of characteristics that people find in their jobs: that is job demands on the one hand and job resources on the other hand. Job demands refer to “those physical, psychological, social, or organizational aspects of the job that require sustained physical, and/or psychological effort and are therefore associated with

For peer review only - http://bmjopen.bmj.com/site/about/guidelines.xhtml

physiological and/or psychological costs".[12] Examples of job demands are workload, time pressure, and emotional demands.[12] High job demands require professionals to spend sustained effort in order to meet perceived demands, which gradually drains resources and ultimately leads to depletion and exhaustion.[20] That being said, job demands are considered the prime factor leading to burnout.[12] Fortunately, professionals also have job resources that support them in coping with job demands. Such job resources, i.e., "physical, psychological, social, or organizational aspects of the job that help to either achieve work goals, reduce job demands and the associated physiological and psychological costs, or stimulate personal growth, learning, and development"[20] can comprise both situational/external and personal/internal resources.[21-22] . Situational resources are, for example, colleague and supervisory support, and the amount of autonomy professionals have in their work.[18,23]. However, resources are not exclusively found in the environment, but people can also create them for themselves.[24] Personal resources refer to individual psychological states such as an individual's psychological capital (including self-efficacy, hope, optimism and resilience), self-compassion (treating oneself with kindness when things go wrong), and cognitive flexibility (the ability to choose behaviors that are in line with ones goals and values) that mirror people's perception to control and impact successfully upon the environment.[25-26]

Apart from supporting employees in coping with job demands, situational and personal resources are also important in their own right as they are considered the prime factor leading to work engagement.[27] Meta-analytic studies have shown, for example, that colleague and supervisory support[13] and optimism and self-efficacy[28] were positively related to work engagement.

The current study builds on the JD-R model that considers burnout and work engagement as independent yet correlated constructs and job demands and (situational and personal) resources as the main predictors of burnout and work engagement, respectively. Although the

For peer review only - http://bmjopen.bmj.com/site/about/guidelines.xhtml

relationships between demands and resources as antecedents and burnout and work engagement as outcomes are confirmed in numerous studies, research also shows that the strength of these relationships varies. This variation is likely due to the different professional samples and work contexts that were studied.[29-31] While residents and specialists work in the same occupational setting, they may face different job characteristics and, hence, different job demands and resources. As such, it is particularly relevant to examine whether the job demands, resources and outcomes, and the relationships among these variables, differ between residents and specialists. Insight into this topic can inform stakeholders how to regulate workplace practices in order to foster physician well-being and work engagement. Importantly, such information can ensure the effectiveness of interventions, because it allows us to tailor interventions to a specific situation or group and tap into personal and situational characteristics that can be changed at the individual and the group level, respectively. In the current study, we therefore investigate how job demands (workload, job insecurity, work-family conflict), situational resources (autonomy, supervisor support, colleague support) and personal resources (psychological capital, self-compassion, psychological flexibility) relate to work engagement and burnout among specialists and residents.

METHOD

Study population

From January to December 2017, we collected data from attending (specialists) and resident physicians at four academic and two general hospitals in the Netherlands. The physicians were specialized or trained in pediatrics, internal medicine, or neurology. This study is part of a larger program offering individual coaching to physicians.

Procedure

All physicians were invited by email to complete an online survey. Participation was voluntary; participants provided informed consent for participation in the study. We took

For peer review only - http://bmjopen.bmj.com/site/about/guidelines.xhtml

measures to safeguard the anonymity and confidentiality of all participants and ethical approval was obtained by the ethical board of the University of Amsterdam.

Measures

To capture the different components of the JD-R model, we included, job demands, job resources, personal resources, burnout and work engagement in the survey, as well as demographics.

Job demands. Job demands were assessed with three scales: workload, job insecurity, and work family conflict.

Workload was assessed with four items from the Quantitative Workload Inventory[32] and two additional items. An example item measuring quantitative workload is: "How often does your job require to work fast?" The two additional items were "How often does your job require you to work overtime? "and "How often do you experience emotional strain from your job?". The items were scored on a five-point scale ranging from 1 ("never") to 5 ("always").

Job insecurity, that is, "the perceived threat of job loss and the worries related to that threat"[33] was measured with an adapted version of the Job Insecurity Scale.[34] The scale consisted of five items including "Chances are, that in the future I won't be able to find the job that I want" or "I am feeling insecure about the future of my career". The items were scored on a seven-point scale ranging from 1 ("not at all applicable") to 7 ("very applicable").

Work-Family Conflict was measured with four items of the Work-Family Conflict Scale measuring the extent to which "the general demands of, time devoted to, and strain created by the work interfere with performing family-related responsibilities".[35] An example item is: "The demands of my work interfere with my home and family life". The items were scored on a seven-point scale ranging from 1 ("not at all applicable") to 7 ("very applicable").

Job resources. Job resources encompassed autonomy, supervisor support, and colleague support.

For peer review only - http://bmjopen.bmj.com/site/about/guidelines.xhtml

Autonomy was measured with nine items from the Work Design Questionnaire[36] assessing perceived autonomy with regard to work scheduling and methods, and decision-making. Example items include “The job allows me to plan how I do my work”, “The job provides me with significant autonomy in making decisions” and “The job allows me to make decisions about what methods I use to complete my work” respectively. The items were scored on a seven-point scale ranging from 1 (“totally disagree”) to 7 (“totally agree”). *Supervisor support,* that is, the experienced psychological and work support from the supervisor was assessed with six items from Vinokur, Schul, and Caplan.[37] Example items include “My supervisor provides me with encouragement” or “My supervisor says things that raise my self-confidence”. For residents, supervisory support measured the support received from the training supervisor, whereas for specialists, supervisory support measured the support received from the head of the department. The items were scored on a seven-point scale ranging from 1 (“totally disagree”) to 7 (“totally agree”).

Colleague support, the experienced psychological and work support from colleagues, was assessed with the same six items as supervisor support, but the items referred to colleagues instead of the supervisor.

Personal resources. We included three personal resources: psychological capital, self-compassion, and psychological flexibility.

Psychological capital was measured with 12 items reflecting hope, optimism, resilience, and efficacy from the validated Dutch version of the PsyCap questionnaire.[38-39] The items include “Right now I see myself as being pretty successful at work” (hope), “I always look on the bright side of things regarding my job” (optimism), “When I have a setback at work, I have trouble recovering from it, moving on (R)” (resilience) and “When encountering difficult problems in my work, I know how to solve them” (efficacy). The items were scored on a seven-point scale ranging from 1 (“totally disagree”) to 7 (“totally agree”).

For peer review only - http://bmjopen.bmj.com/site/about/guidelines.xhtml

For peer review only

Self-compassion, entailing "treating oneself with kindness, recognizing one's shared humanity, and being mindful when considering negative aspects of oneself"[40] was measured with six items from the Self-Compassion Scale.[40] Example items are: "When I am going through a very hard time, I give myself the caring and tenderness I need" (self-kindness), "I try to see my failings as part of the human condition" (common humanity), and "When something painful happens I try to take a balance view of the situation" (mindfulness). The items were scored on a five-point scale ranging from 1 ("rarely") to 5 ("almost always").

Psychological flexibility, that is, the ability to flexibly take appropriate action towards achieving goals and values, even in the presence of challenging or unwanted events[41] was measured with the Work Acceptance and Action Questionnaire.[42] Example items include "I am able to work effectively in spite of any personal worries that I have" and "I can work effectively, even when I doubt myself." The items were scored on a five-point scale ranging from 1 ("rarely") to 5 ("almost always").

Outcomes. The outcome variables included in this study were burnout symptoms and work engagement.

Burnout was measured with the Dutch version[43] of the Maslach Burnout Inventory – General Survey[44] [a]. *Exhaustion* was measured with 5 items. An example item is: "Working all day is really a strain for me." The items were scored on a seven-point scale ranging from 1 ("totally disagree") to 7 ("totally agree"). *Cynicism* was measured with 4 items. An example item is: "I noticed that I have got too much distance from my work." The items were scored on a seven-point scale ranging from 1 ("totally disagree") to 7 ("totally agree").

Work engagement, including vigor, dedication, and absorption at work, was measured with nine items from the Utrecht Work Engagement Scale.[46] Example items include: "When I get up in the morning, I feel like going to work" (vigor), "I am enthusiastic about my job"

[a] The instrument consists of three subscales measuring exhaustion, cynicism and professional efficacy. Because exhaustion and cynicism constitute the essence of the burnout syndrome[45], we only measured these two components.

For peer review only - http://bmjopen.bmj.com/site/about/guidelines.xhtml

(dedication), and "When I am working I forget everything around me" (absorption). The items were scored on a seven-point scale ranging from 1 ("never") to 7 ("always").

Statistical analysis

Between group variance. Because participants can be considered as nested within (four) hospitals and (three) specializations, we first assessed between group variance within our data. A multilevel mixed-method analysis estimating a random intercept model was conducted to calculate between level-2 variance[b].

Control variables. We explored the association between potential control variables (i.e., age, gender, having children, job tenure, signed up for coaching) and the dependent variables by means of regression analyses for residents and specialists separately.

Path analysis. The relationships between the independent (job demands, job resources, and personal resources) and dependent variables (exhaustion, cynicism, and work engagement), were examined with path analysis using IBM SPSS AMOS 25 (IBM SPSS, Chicago). In a first step, we modeled a latent variable termed burnout based on the observed variables exhaustion and cynicism. Modeling these two outcome variables on one latent variable was justified both theoretically[45,47] and statistically (correlation of $r = .58$, $p < .01$ between exhaustion and cynicism).

A path model with independent variables and work engagement and burnout as dependent variables was tested using a covariance matrix as input and maximum likelihood estimation. This analysis adequately captures the nature of the associations between the independent and dependent variables and was therefore chosen over regular OLS regression analyses. Furthermore, this analysis allowed for a multi-group comparison, testing possible differences in model estimates between residents and specialists. We report the commonly used model fit criteria, the chi-square goodness-of-fit value, the chi-square divided by the degrees of

[b] The final Hessian matrix was not positive definite as the intercept variance was zero.

For peer review only - http://bmjopen.bmj.com/site/about/guidelines.xhtml

freedom (CMIN/DF), the comparative fit index (CFI), the root mean square error of approximation (RMSEA) and the standardized root mean squared residual (SRMR).

Patient and public involvement

This study investigated factors associated with work engagement and burnout in medical specialists and residents. No patients or public representatives were involved in the study.

RESULTS

In total, we invited a number of 247 physicians to take part in this survey of whom 75 physicians had signed up for a personal coaching program that would start in a few months. A total number of *N* = 193 physicians participated in this study (response rate = 78 %). The study population included 151 females (78.2 %) and 42 males (21.8 %). The mean age was 36.5 years (*SD* = 8.5). 124 residents (64.2 %) and 69 medical specialists (35.8 %) participated. Participants were working in the field of pediatrics (*n* = 142; 73.6 %), neurology (*n* = 14; 7.3 %), and internal medicine (*n* = 37; 19.2 %). See table 1 for a description of participants' characteristics. Internal consistency (Cronbach alpha) was acceptable for all scales (see Table 2).

For peer review only - http://bmjopen.bmj.com/site/about/guidelines.xhtml

Table 1

Demographics of Residents and Specialists Participating in a Study on the Role of Personal Resources for Work Engagement and Burnout, 2017[a]

Characteristics	Residents	Specialists
	No. (% of 124)	No. (% of 69)
**Gender**		
Female	101 (81.5%)	50 (72.5%)
Male	23 (18.5%)	19 (27.5)
**Age[b]**		
20 - 30 years	42 (33.9%)	1 (1.4%)
31 - 40 years	81 (65.3%)	28 (40.6%)
41 - 50 years	1 (0.8%)	22 (31.9%)
51 - 60 years	-	15 (21.7%)
61 years and older	-	3 (4.3%)
**Specialty**		
Pediatrics	87 (70.2%)	55 (79.7%)
Internal medicine	27 (21.8%)	10 (14.5%)
Neurology	10 (8.1%)	4 (5.8%)
**Signed up for coaching[c]**		
Yes	53 (42.7%)	36 (52.2%)
No	71 (57.3%)	33 (47.8%)
**Home situation**		
Children, one or more	51 (41.1%)	48 (69.6%)
No children	73 (58.9%)	21 (30.4%)

[a]This study was conducted at 4 academic and 2 general hospitals in The Netherlands. In this study, the authors investigated associations between job demands (workload, work-family conflict, job insecurity), job resources (autonomy, supervisor support, colleague support), personal resources (psychological capita, self-compassion, psychological flexibility) with work engagement and burnout.
[b]The residents taking part in this study were on average 31.9 years old, (standard deviation [SD] = 3.0); the specialists were on average 44.9 years old (SD = 8.7).
[c]Participants were registered to participate in an institutional coaching program or private coaching.

For peer review only - http://bmjopen.bmj.com/site/about/guidelines.xhtml

Table 2

Correlations between the Study Variables for Residents and Specialists and Internal Consistencies[c] of Study Variables in a Study on the Role of Personal Resources for Work Engagement and Burnout, 2017[a-b]

**Study Variables**	**1**	**2**	**3**	**4**	**5**	**6**	**7**	**8**	**9**	**10**	**11**	**12**	**13**	**14**
1. Signed up for coaching[d]	-	.11	.31*	-.27*	-.40**	.18	.02	.32**	.28*	.23	.16	-.26*	-.11	.05
2. Job tenure	.13	-	.02	-.22	-.10	.14	-.05	.11	.19	.21†	.00	.12	.10	.02
3. Workload	-.09	.08	**0.85**	.18	.46**	-.14	-.00	-.16	-.14	-.16	.06	.33**	.27*	.02
4. Job insecurity	-.16†	.18*	.23*	**0.83**	.10	-.29*	-.27*	-.21†	-.53**	-.42**	-.29*	.29*	.38**	-.41**
5. Work-family conflict	-.13	.11	.36**	.15	**0.87**	-.32**	-.16	-.10	-.25*	-.36**	.01	.42**	.27**	-.07
6. Autonomy	.13	.13	-.29**	-.18*	-.11	**0.93**	.21	.29*	.55**	.37**	.18	-.35**	-.28*	.38**
7. Colleague support	.08	-.03	-.19*	-.24**	-.06	.18*	**0.94**	.08	.43**	.47**	.16	-.47**	-.58**	.60**
8. Supervisor support	-.07	-.17	.01	-.07	.14	.20*	.08	**0.95**	.41**	.25*	.31**	-.15	-.16	.25*
9. PsyCap	.13	-.13	-.22*	-.38**	-.21*	.33**	.26**	.19*	**0.88**	.68**	.37**	-.50**	-.60**	.58**
10. Self-compassion	.13	-.13	-.32**	-.41**	-.33**	.15	.22*	.09	.51*	**0.72**	.25*	-.33**	-.50**	.32**
11. Psych. flexibility	.06	.03	-.01	-.13	-.02	.30**	.26**	.05	.37*	.13	**0.81**	.10	-.17	.32**
12. Exhaustion	-.05	.15†	.35**	.22*	.51**	-.04	-.23*	-.07	-.32*	-.45**	-.26**	**0.84**	.67**	-.50**
13. Cynicism	.14	.26**	.23**	.23*	.23*	-.21*	-.33**	-.17	-.42**	-.26**	-.37**	.58**	**0.77**	-.67**
14. Work engagement	-.05	-.04	-.12	-.15	-.14	.31**	.24**	.20*	.54**	.29**	.38**	-.40**	-.62**	**0.90**

Abbreviations: PsyCap indicates psychological capital; psych. flexibility indicates psychological flexibility.
[a]The values below the diagonal refer to residents. The values above the diagonal refer to specialists.
bStatistical significance of correlation coefficients is indicated by the following symbols:†p < .10. *p < .05. ** p < .01.
[c]Internal consistencies (Cronbach alpha) are displayed on the diagonal in bold numbers.
[d]Participation (yes) was indicated with the number 1, participation (no) was indicated with the number 2.

For peer review only - http://bmjopen.bmj.com/site/about/guidelines.xhtml

Between group variance

A multivariate GLM analysis confirmed that hospitals and specializations did not significantly differ on exhaustion, cynicism, and work engagement. Therefore, it was not necessary to account for group level effects when estimating the relationships between the independent and dependent variables.

Control variables

The results showed that only the control variables job tenure; $b = .23$, $p = .02$ (related to exhaustion for residents), $b = .24$, $p = .02$ (related to cynicism for residents) and signed up for coaching[c]; $b = -.31$, $p = .01$ (related to exhaustion for specialists) were related to exhaustion, cynicism, or engagement. Therefore, and to save power, we only included job tenure and signed up for coaching as control variables in the further analyses.

Descriptives and group differences

Table 3 describes means, standard deviations and differences in study variables for residents and specialists, respectively. Independent sample t-tests were performed to investigate mean-level differences in study variables comparing residents and specialists. Compared to specialists, residents reported significantly lower workload ($M = 3.29$, $SD =.68$ vs. $M = 3.52$ $SD = .83$, $p^d < .05$), lower autonomy ($M = 4.10$, $SD =.99$ vs. $M = 5.19$, $SD = 1.02$, $p < .01$), and lower work engagement ($M = 4.93$, $SD = .77$ vs. $M = 5.21$ $SD = .88$, $p < .05$). However, residents reported significantly higher job insecurity than specialists ($M = 4.26$, $SD = 1.17$ vs. $M = 3.03$, $SD = 1.27$, $p < .01$).

[c] Response options were: 1 = yes, 2 = no
[d] Significance values were $p = .039$ when equal variances were assumed, and $p = .052$ when equal variances were not assumed.

For peer review only - http://bmjopen.bmj.com/site/about/guidelines.xhtml

Table 3

Means and Standard Deviations (SD) of Study Variables for Residents and Specialists in a Study on the Role of Personal Resources for Work Engagement and Burnout, 2017[c]

Study Variables	Residents[a]	Specialists[b]
	Mean (SD)	Mean (SD)
**Work demands**		
Workload*[d]	3.29 (.68)	3.52 (.83)
Job insecurity**	4.26 (1.17)	3.03 (1.27)
Work-family conflict	4.47 (1.14)	4.56 (1.32)
**Job resources**		
Autonomy**	4.10 (.99)	5.19 (1.02)
Colleague support	5.37 (.85)	5.38 (1.17)
Supervisor support	4.66 (1.39)	4.84 (1.49)
**Personal resources**		
PsyCap[†]	4.91 (.72)	5.11 (.75)
Self-compassion[†]	3.19 (.63)	3.36 (.65)
Psych. flexibility	3.53 (.61)	3.62 (.62)
**Outcomes**		
Exhaustion	2.55 (1.02)	2.40 (1.18)
Cynicism	2.24 (.99)	2.01 (1.07)
Work engagement*	4.93 (.77)	5.21 (.88)

Abbreviation: SD indicates standard deviation; PsyCap indicates psychological capital; psych. flexibility indicates psychological flexibility.
[a]A total number of 124 residents participated.
[b]A total number of 69 specialists participated.
[c]Differences in means between residents and specialists are indicated by the following significance values: [†]$p < .10$; *$p < .05$; **$p < .01$.
[d]Significance values were $p = .039$ with equal variances assumed, and $p = .052$ with equal variances not assumed.

For peer review only - http://bmjopen.bmj.com/site/about/guidelines.xhtml

Path analyses

Preliminary analyses. As suggested by Joreskög and Sörbom[48], we first specified an initial model based on our research question, and then adjusted the model according to the modification indices it produced, allowing covariation between all predictor variables, as well as covariation between job tenure and autonomy. Because both indicators of burnout highly correlated with engagement, we allowed covariation of error variance between exhaustion and cynicism with engagement. Testing the initial path model for specialists and residents separately revealed that the control variable signed up for coaching was not related to any of the two outcomes in both sub-samples. We, therefore, removed this variable from the analysis and continued the analysis with only job tenure as control variable.

Model fit. The path analysis showed a satisfactory fit to the data, $X^2 = 109.25$ (51), $p < .001$, $X^2/\text{df} = 2.14$, CFI = .96, RMSEA = .06, SRMR = .06. In order to improve the model fit, we removed the paths that were non-significant for both residents and specialists.[49]

We removed the paths from job insecurity and supervisor support to burnout. Further, we removed the paths from workload, job insecurity, work-family conflict, autonomy, supervisor support and job tenure to work engagement as they were not significant, partly despite significant zero-order correlations between these variables (see Table 2). The model resulted in an improved fit of $X^2 = 132.33$ (75), $p < .001$, $X^2/\text{df} = 1.76$, CFI = .96, RMSEA = .05, SRMR = .06. The tested model is presented in Figure 1. The model explained 53.9 % of the variance in burnout, and 27.9 % of variance in work engagement.

Relationships with burnout. The standardized path coefficients with burnout as outcome are presented in Table 4.

For peer review only - http://bmjopen.bmj.com/site/about/guidelines.xhtml

Table 4

Standardized Parameter Estimates and Group Comparison of the Paths Across Residents and Specialists in a Study on the Role of Personal Resources for Work Engagement and Burnout, 2017[a-c]

	Parameter estimate		
Path	Residents	Specialists	Group comparison[d]
Workload → Burnout	.20*	.22**	.929
Work-family conflict → Burnout	.33***	.15†	.135
Autonomy → Burnout	.19*	.10	.465
Colleague support → Burnout	-.12	-.41***	.088†
PsyCap → Burnout	-.09	-.58***	.003**
Self-compassion → Burnout	-.22*	.07	.072†
Psych. flexibility → Burnout	-.31***	.06	.003**
Colleague support → Work Engagement	.03	.49***	.001**
PsyCap → Work Engagement	.37***	.55***	.135
Self-compassion → Work Engagement	.11	-.33**	.003**
Psych. flexibility → Work Engagement	.17*	.11	.778

Abbreviation: PsyCap indicates psychological capital; psych. flexibility indicates psychological flexibility; the direction of the paths are indicated by an arrow symbol.
[a]Burnout as latent variable is based on the observed variables exhaustion and cynicism.
[b]Only significant paths are displayed; the paths from job insecurity and supervisor support to burnout were not significant. Also the paths from work-family conflict, autonomy, supervisor support, job insecurity and workload to work engagement were not significant.
[c]Statistical significance of parameter estimates and group comparison is indicated by the following symbols: †$p < .10$. *$p < .05$. ** $p < .01$. ***$p < .001$.
[d]This column depicts significance values associated with possible differences in model estimates between residents and specialists.

Job demands. Separate tests for residents and specialists suggested that there were no differences between both groups: workload was positively related to burnout for residents ($b = .20$, $p = .011$) and specialists ($b = .22$, $p = .009$). A multi-group comparison test confirmed that these relationships did not significantly differ, $p > .05$. Furthermore, separate tests for both groups suggested that work-family conflict as a job demand differed between residents and specialists: it was positively related to burnout for residents ($b = .33$, $p < .001$) but not for

For peer review only - http://bmjopen.bmj.com/site/about/guidelines.xhtml

For peer review only

specialists ($p > .05$). However, these relationships did not significantly differ in a multi-group comparison test, $p > .05$.

Job resources. Separate tests for both groups suggested that there were differences between residents and specialists regarding autonomy: autonomy was positively related to burnout for residents ($b = .19$, $p = .016$), but not for specialists ($p > .05$). However, these differences did not significantly differ in a multi-group comparison test. Furthermore, separate tests for both groups suggested that colleague support differed between residents and specialists: it was not related to burnout for residents ($p > .05$), but was negatively related to burnout for specialists ($b = -.41$, $p < .001$). However, these relationships did not significantly differ in a multi-group comparison test, $p = .088$.

Personal resources. Separate tests for both groups suggested that there were differences between residents and specialists regarding the personal resource psychological capital: psychological capital was not related to burnout for residents ($p > .05$), but was negatively related to burnout for specialists ($b = -.58$, $p < .001$). A multi-group comparison test confirmed that these relationships significantly differed, $p = .003$. Furthermore, separate tests for both groups suggested that self-compassion differed between residents and specialists: self-compassion was negatively related to burnout for residents ($b = -.22$, $p = .017$) but not related to burnout for specialists, $p > .05$. However, these relationships did not significantly differ in a multi-group comparison test, $p = .072$. Also, separate tests suggested that there were differences between residents and specialists regarding the personal resource psychological flexibility: it was related to burnout for residents ($b = -.31$, $p < .001$), but not related to burnout for specialists ($p > .05$). A multi-group comparison test confirmed that these relationships did significantly differ, $p = .003$.

Relationships with work engagement. The standardized path coefficients with work engagement as outcome are presented in Table 4.

For peer review only - http://bmjopen.bmj.com/site/about/guidelines.xhtml

Job resources. Separate tests suggested that there were differences regarding colleague support between residents and specialists: colleague support was not related to work engagement for residents ($p > .05$), but was positively related to work engagement for specialists ($b = .49$, $p < .001$). A multi-group comparison test confirmed that these relationships did significantly differ, $p = .001$.

Personal resources. Separate tests for both groups suggested that there were no differences between residents and specialists regarding psychological capital: psychological capital was positively related to work engagement for both residents ($b = .37$, $p < .001$) and specialists ($b = .55$, $p < .001$). A multi-group comparison test confirmed that these relationships did not significantly differ, $p > .05$. Furthermore, separate tests for both groups suggested that there were differences between residents and specialists regarding self-compassion: self-compassion was not related to work engagement for residents ($p > .05$), but was negatively related to work engagement for specialists ($b = -.33$, $p = .004$). A multi-group comparison test confirmed that these relationships did significantly differ, $p =.003$. Also, separate tests for both groups suggested that there were differences between residents and specialists regarding psychological flexibility: psychological flexibility was positively related to work engagement for residents ($b = .17$, $p = .035$), but not for specialists ($p > .05$). However, a multi-group comparison test showed that these relationships did not significantly differ, $p = .778$.

DISCUSSION

Main findings

The goal of this study was to gain insight on the prevailing demands and resources that contribute to burnout and work engagement in medical residents and specialists. This study revealed that residents and specialists face different demands in their work that cannot be measured by the same yardstick but instead require tailored solutions. Confirming prior studies on the stressful demands during residency,[7] our data showed that residents as

For peer review only - http://bmjopen.bmj.com/site/about/guidelines.xhtml

compared to specialists experienced less autonomy and felt more uncertain about the future of their job. Furthermore, symptoms of exhaustion and cynicism among residents increased with tenure, which may at least partly relate to growing feelings of job insecurity (see Table 2). Contrary to what has been reported in previous studies,[50] residents did not report significantly higher exhaustion and cynicism than specialists but, on average, felt less engaged with their work.

We suggest that specialists and residents resort to different resources to cope with their job demands. While specialists benefit from psychological capital and colleague support, residents benefit especially from psychological flexibility and self-compassion. In addition, psychological capital was found to play a role for the work engagement of both specialists and residents, which corroborates earlier findings among other professional groups.[51] Physicians are exposed to high job demands, both during attendance and residency, which could harm their well-being.[12] However, our study suggests that job demands other than workload (e.g. job insecurity, work-family conflict) and a lack of resources (e.g., self-compassion, psychological capital, and psychological flexibility) play a prominent role in the onset of burnout. The fact that the residents in this study reported a relatively lower workload than specialists, yet reported similar symptoms of burnout, underlines this notion. Generally, our findings suggest that for preventing burnout it is important to focus on those demands and resources that are most relevant for specific groups of physicians (e.g., specialists or residents). Specialists may particularly benefit from interventions that raise their psychological capital and – at the team level – foster team cohesion and support whereas residents may benefit relatively more from interventions that increase their self-compassion and flexibility.

Resources that buffer burnout among specialists and residents

For peer review only - http://bmjopen.bmj.com/site/about/guidelines.xhtml

Personal resources. Consistent with prior research,[52-53] psychological capital played an eminent role for the well-being of specialists. Psychological capital may reduce the risk of burnout in two ways. First, it can counteract the distress associated with a demanding workplace by regulating negative emotions.[54] Second, individuals with high psychological capital tend to perceive job demands as challenges rather than hindrances. That is, they associate job demands with personal gain or growth,[55] evoking positive emotions, instead of fear and threat, evoking negative emotions.[55-56] Given the buffering capacity of psychological capital,[52-53] it is surprising that we did not find the same result among residents. Instead, we found that flexibility and self-compassion rather than psychological capital contributed to the well-being of residents.

The importance of flexibility and self-compassion among residents may be due to their specific career phase, which is characterized by insecurity, constant feedback, and criticism. That is, residency is an extremely challenging period, in which residents have to deal with their newly gained responsibilities (i.e., managing uncertainty, breaking bad news) whilst also processing new information and continuously adapting to new organizational structures and teams. Residents have to shift regularly between their roles as trainee and doctor,[57] experience high challenges at work, and are confronted with their relative lack of knowledge and skills when entering residency. More so, residents are taught from medical school on to be critical towards themselves, a necessity that is demanded in a high-stake work environment where carelessness can have radical consequences. Consequently, medical professionals likely adopt a rather self-critical attitude.[58] One way to deal with these stressful work events is to accept one's inexperience, forgive one's deficiencies (i.e., self-compassion) and remain effective despite self-doubt and worries (i.e., psychological flexibility). More specifically, residents need to internalize that despite the current healthcare culture, not-knowing, insecurities and mistakes are part of the journey and not a sign of weakness or

For peer review only - http://bmjopen.bmj.com/site/about/guidelines.xhtml

failure. In addition, psychological flexibility may allow residents to shift between tasks and professional roles, as it facilitates adapting to fluctuating situational demands, shifting perspective, and reconfiguring mental resources.[59] Thus, being kind towards oneself and viewing one's own shortcomings as human can help to safeguard residents against the stressors uniquely present in residency.

Colleague support. In addition to psychological capital, colleague support also seems to promote the well-being of specialists. Numerous studies have indeed shown that social support is associated with both psychological and physical health outcomes[60-61] as it ameliorates the impact of stress and strain on health.[23,61-62] First, social support may involve emotional support, the feeling that one is loved and cared for.[63] Secondly, it may involve the provision and sharing of information.[63] Specialists work in relatively permanent teams with interdependent work relationships. Knowing their colleagues and their expertise well, they can ask for and receive emotional but also informational support. Colleagues can provide intimacy or reassurance during emotional and stressful events and they can assist in times of uncertainty and difficult medical inquiries that require another expert opinion.

Although our data does not allow any insights in the quality of support received from colleagues, it is possible that the quality of support is different for residents and specialists. Because of the nature of residency (e.g., competition and regular rotations) it is likely that colleague relationships are relatively less permanent and fruitful for residents. Although valuing their opinion, residents might not be convinced that they can ultimately lean on a fellow residents' opinion in solving medical problems. This could explain why residents benefit relatively less from colleague support than specialists.

Resources that foster work engagement among specialists and residents

For peer review only - http://bmjopen.bmj.com/site/about/guidelines.xhtml

Personal resources. Our finding that psychological capital is a personal resource that is vital for the work engagement of both residents and specialists corroborates with prior studies among other professional groups.[54-55]

Unexpectedly, self-compassion was negatively rather than positively associated with the work engagement of specialists, while we found no such effect for residents. It is possible that high levels of self-compassion represent a self-protective bias, which serves to deny responsibility for failure.[64] That is, high self-compassion may lean towards attributing failure to external factors (e.g., situational constrains, lack of help from others) allowing specialists to maintain positive perceptions of their capabilities. Over a longer period of time, this way of thinking about their own shortcomings may hamper specialists' personal development and work efforts, which may ultimately cause a reduction in work engagement. It is therefore worth exploring whether the benefits of self-compassion depend on time, or whether there is an optimal level - or perhaps a tipping point - at which self-compassion contributes to one's well-being.

Colleague support. Colleague support not only buffered the occurrence of burnout, but also fostered work engagement among specialists rather than residents. As argued above, specialists as opposed to residents work in more permanent teams. It has been consistently found that the social support in these teams facilitates the work engagement of team members.[65]

Study strengths and limitations

The participation of both residents and specialists from different specialties and the high response rate (78 %) allowed for a realistic display of the demands and resources that physicians encounter during different stages of their career. Our research shows how different demands and resources relate to burnout and work engagement among specific groups of physicians, and, as such, advances our understanding of how to intervene when well-being or

For peer review only - http://bmjopen.bmj.com/site/about/guidelines.xhtml

work engagement are at risk. This is an important first step in the prevention of burnout and the conservation of work engagement among healthcare professionals. Yet, our results also indicate that burnout and work engagement are highly interweaved, just as in numerous prior studies. [65-66] Consequently, it is important to consider both burnout and work engagement when addressing job functioning as a whole.

Finally, our study has some limitations. Firstly, although theory and research point to causal relationships between demands and burnout and resources and work engagement,[12,67] our design does not allow to draw causal conclusions. It is possible that the proposed relationships in the JD-R model are reversed. For instance, feelings of exhaustion and cynicism may change the way employees perceive their work demands, intensifying the feeling that demands are piling up.[68-69] Future research could use multi-wave designs that can provide insight into the development of study variables over time and the causal dynamics in this process[70]. Secondly, all data have been gathered using self-report questionnaires. This might lead to a so-called 'common-method bias'.[71] A potential way to reduce this bias would be to expand the sources of information (e.g., supervisors' assessments of employee burnout and engagement) and the methods of data collection (i.e., triangulation of data), for instance by including qualitative data as a next step. We are aware that self- and observer ratings of work characteristics and job demands may not necessarily correlate high[12]. However, we believe that expanding the information source through third-party observations as well as triangulation can help to provide a richer picture of the work characteristics being studied. Finally, the concept of colleague support is limited in the sense that it does not allow for a differentiation between support functions. Ideally, a concept of social support including such a differentiation[63] could help to disentangle *how* perceived social support helps specialists to counteract stress and exhaustion and promote work engagement.

Implications

For peer review only - http://bmjopen.bmj.com/site/about/guidelines.xhtml

To our knowledge, this is the first study that attempts to reveal the specific demands and resources that may impact burnout and work engagement among residents and specialists. Understanding how demands and resources are linked to physician well-being and engagement is a fundamental premise for designing successful interventions to minimize the risk of burnout. Our results suggest that a one-size-fits-all approach might not be effective for promoting physician well-being but, instead, that interventions should be tailored to the specific needs of specialists and residents.[2] This is in line with a recent call to consider contextual complexities such as specialty or career stage when setting up interventions to promote physician well-being.[19] While interventions for specialists should focus on increasing psychological capital and colleague support, interventions for residents should, in addition to increasing psychological capital, be aimed at increasing, self-compassion and psychological flexibility. Interestingly, especially personal resources seemed to preserve physician well-being and engagement. Therefore, targeting personal resources rather than structural constraints seems promising to counter the demands physicians face. We consider testing the effectiveness of interventions aiming at fostering personal resources an important future inquiry.

CONCLUSION

With physician well-being being central to optimal patient care, it is important to uncover work characteristics that influence work engagement and burnout. This study revealed that physicians are not a uniform body, but that medical residents and specialists face different challenges in their work that require unique resources to resort to. While all physicians are likely to benefit from resources facilitating goal attainment (i.e., psychological capital), medical residents may additionally benefit from self-care and flexibility, and specialists may additionally benefit from social support. Finally, by respecting also the unique needs of

For peer review only - http://bmjopen.bmj.com/site/about/guidelines.xhtml

residents and specialists, one can create equal opportunities for all physicians in the challenging workplace that healthcare is.

For peer review only

For peer review only - http://bmjopen.bmj.com/site/about/guidelines.xhtml

Acknowledgments

The authors wish to thank all the residents and attending physicians who participated in this study. The authors also want to thank Axel Willemse, Sarah van den Hee, dr. Machteld van den Heuvel, and dr. Angelique Bakker-Pieper for their contribution to the design and implementation of the study.

Contributors

All authors were involved in the conception and design of the study as well as the collection and interpretation of the data. LS, AVV, and JK analyzed the data. All authors reviewed and approved the manuscript.

Funding/Support

This research received no specific grant from any funding agency in the public, commercial or not-for-profit sectors.

Competing interests

None declared.

Ethical approval

The institutional Ethic Review Board of the University of Amsterdam waived ethical approval for this study, on December 12, 2016; document 2016-WOP-7521.

Disclaimer

None reported.

Data sharing statement

Data and statistical code are available upon request. Any queries should be directed to the first author.

Previous presentations

This research has been presented at the annual national conference of the Netherlands Association for Medical Education (NVMO) in Egmond aan Zee, The Netherlands (November 16, 2018).

For peer review only

For peer review only - http://bmjopen.bmj.com/site/about/guidelines.xhtml

REFERENCES

1. Schaufeli WB, Salanova M, González-Romá V, Bakker AB. The measurement of engagement and burnout: A two sample confirmatory factor analytic approach. J Happiness Stud. 2002; 3: 71-92.
2. Scheepers RA, Lases LS, Arah OA, Heineman MJ, Lombarts KM. Job resources, physician work engagement, and patient care experience in an academic medical setting. Acad Med. 2017; 92: 1472-1479.
3. Thomas NK. Resident burnout. JAMA. 2004; 292: 2880-2889.
4. Firth-Cozens J. Doctors, their wellbeing, and their stress: It's time to be proactive about stress - and prevent it. BMJ. 2003; 326: 670.
5. Maslach C, Jackson SE. The measurement of experienced burnout. J Organ Behav. 1981; 2: 99-113.
6. Shanafelt TD, Hasan O, Dyrbye L, et al. Changes in burnout and satisfaction with work-life balance in physicians and the general US working population between 2011 and 2014. Mazo Clin Proc. 2015; 90: 1600-1613.
7. Dyrbye LN, Varkey P, Boone SL, Satele DV, Sloan JA, Shanafelt TD. Physician satisfaction and burnout at different career stages. Mayo Clin Proc. 2013; 88: 1358-1367.
8. Prins JT, Hoekstra-Weebers JEHM, Van De Wiel HBM, et al. Burnout among Dutch medical residents. Int J Behav Med. 2007; 14: 119-125.
9. Shanafelt TD, Bradley KA, Wipf JE, Back AL. Burnout and self-reported patient care in an internal medicine residency program. Ann Intern Med. 2002; 136: 358-367.
10. Rimmer A. Employers must tackle high level of burnout among trainees, says GMC. BMJ. 2018; 362: k3018.
11. Campbell J, Prochazka AV, Yamashita T, Gopal R. Predictors of persistent burnout in internal medicine residents: a prospective cohort study. Acad Med. 2010; 85: 1630-1634.

For peer review only

For peer review only - http://bmjopen.bmj.com/site/about/guidelines.xhtml

12. Demerouti E, Bakker AB, Nachreiner F, Schaufeli WB. The job demands-resources model of burnout. J Appl Psychol. 2001; 86: 499-512.

13. Christian MS, Garza AS, Slaughter JE. Work engagement: A quantitative review and test of its relations with task and contextual performance. Pers Psychol. 2011; 64: 89-136.

14. Salanova M, Agut S, Peiró JM. Linking organizational resources and work engagement to employee performance and customer loyalty: the mediation of service climate. J Appl Psychol. 2005; 90: 1217-1227.

15. Loerbroks A, Glaser J, Vu-Eickmann P, Angerer P. Physician burnout, work engagement and the quality of patient care. Occup Med. 2017; 67: 356-362.

16. Prins JT, Van Der Heijden FMMA, Hoekstra-Weebers JEHM, et al. Burnout, engagement and resident physicians' self-reported errors. Psychol Health Med. 2009; 14: 654-666.

17. Maslach C, Leiter MP. The truth about burnout: how organizations cause personal stress and what to do about it. San Francisco. CA: Jossey-Bass; 1997

18. Schaufeli WB, Bakker AB. Job demands, job resources, and their relationship with burnout and engagement: A multi-sample study. J Org Behav. 2004; *25*: 293-315.

19. Carrieri D, Briscoe S, Jackson M, et al. 'Care Under Pressure': a realist review of interventions to tackle doctors' mental ill-health and its impacts on the clinical workforce and patient care. BMJ Open, 2018; 8: e021273.

20. Bakker AB, Demerouti E. The job demands-resources model: State of the art. J Manage Psychol. 2007; 22: 309-328.

21. Xanthopoulou D, Bakker AB, Demerouti E, Schaufeli WB. Reciprocal relationships between job resources, personal resources, and work engagement. J Vocat Behav. 2009; *74*: 235-244.

22. Richter P, Hacker W. Workload and strain: Stress, fatigue, and burnout in working life. Heidelberg, Germany: Asagner; 1998.

For peer review only

For peer review only - http://bmjopen.bmj.com/site/about/guidelines.xhtml

23. Bakker AB, Demerouti E, Euwema MC. Job resources buffer the impact of job demands on burnout. J Occup Health Psych. 2005; 10:170-180.

24. Hobfoll SE. Conservation of resources: A new attempt at conceptualizing stress. Am Psychol. 1989*;* 44: 513-524.

25. Hobfoll SE, Johnson RJ, Ennis N, Jackson AP. Resource loss, resource gain, and emotional outcomes among inner city women. J Pers Soc Psychol. 2003; *84*: 632-643.

26. Xanthopoulou D, Bakker AB, Demerouti E, Schaufeli WB. The role of personal resources in the job demands-resources model. Int J Stress Manag. 2007; 14: 121-141.

27. Hakanen JJ, Bakker AB, Schaufeli WB. Burnout and work engagement among teachers. J Sch Psychol. 2006; 43: 495-513.

28. Halbesleben JR. A meta-analysis of work engagement: Relationships with burnout, demands, resources, and consequences. Work engagement: A handbook of essential theory and research. 2010; 8: 102-117.

29. Murray MA, Cardwell C, Donnelly M. GPs' mental wellbeing and psychological resources: a cross-sectional survey. Br J Gen Pract. 2017; 67: e547-e554

30. Van den Heuvel M, Demerouti E, Bakker AB, Schaufeli WB. Personal resources and work engagement in the face of change. In: Houdmont J, Leka S. Contemporary occupational health psychology: Global perspectives on research and practice. Hoboken, New Jersey: John Wiley & Sons; 2010: 124-150.

31. Crawford ER, LePine JA, Rich BL. Linking job demands and resources to employee engagement and burnout: a theoretical extension and meta-analytic test. J Appl Psychol. 2010; 95: 834-848.

32. Spector PE, Jex SM. Development of four self-report measures of job stressors and strain: interpersonal conflict at work scale, organizational constraints scale, quantitative

For peer review only - http://bmjopen.bmj.com/site/about/guidelines.xhtml

workload inventory, and physical symptoms inventory. J Occup Health Psychol, 1998; 3: 356-367.

33. De Witte H. Job insecurity: Review of the international literature on definitions, prevalence, antecedents and consequences. SA J Ind Psychol. 2005; 31:1-6.

34. De Witte H. Arbeidsethos en jobonzekerheid: Meting en gevolgen voor welzijn, tevredenheid en inzet op het werk [Work ethics and job insecurity: Measurement and consequences for well-being, satisfaction, and work engagement]. In: Bouwen R, De Witte K, De Witte H, Taillieu T. Van groep naar gemeenschap. Liber Amicorum Prof. Dr. Leo Lagrou. Leuven, Belgium: Garant. 2000:325-350.

35. Netemeyer RG, Boles JS, McMurrian R. Development and validation of work–family conflict and family–work conflict scales. J Appl Psychol. 1996; 81:400-410.

36. Morgeson FP, Humphrey SE. The Work Design Questionnaire (WDQ): developing and validating a comprehensive measure for assessing job design and the nature of work. J Appl Psychol. 2006; 91:1321-1329.

37. Vinokur A, Schul Y, Caplan RD. Determinants of perceived social support: Interpersonal transactions, personal outlook, and transient affective states. J Pers Soc Psychol. 1987; 53:1137-1145.

38. Ouweneel E, Le Blanc PM, Schaufeli WB. Don't leave your heart at home: Gain cycles of positive emotions, resources, and engagement at work. Career Dev Int. 2012; 17:537-556.

39. Luthans F, Youssef CM, Avolio BJ. Psychological capital: Developing the human competitive edge. Oxford: Oxford University Press; 2007.

40. Neff KD, Vonk R. Self-compassion versus global self-esteem: Two different ways of relating to oneself. J Pers. 2009; *77*:23-50.

41. Hayes SC, Luoma JB, Bond FW, Masuda A, Lillis J. Acceptance and commitment therapy: Model, processes and outcomes. Behav Res Ther. 2006; *44*:1-25.

For peer review only - http://bmjopen.bmj.com/site/about/guidelines.xhtml

42. Bond FW, Lloyd J, Guenole N. The work-related acceptance and action questionnaire: Initial psychometric findings and their implications for measuring psychological flexibility in specific contexts. J Occup Organ Psychol. 2013; *86*:331-347.

43. Schaufeli WB, van Dierendonck D. UBOS Utrechtse Burnout Schaal: Handleiding. Lisse, The Netherlands: Swets Test Publishers; 2000.

44. Maslach C, Jackson SE, Leiter MP, Schaufeli WB, Schwab RL. Maslach burnout inventory. Palo Alto, CA: Consulting Psychologists Press; 1986; 21: 3463-3464.

45. Schaufeli WB, Buunk BP. Burnout: An overview of 25 years of research and theorizing. In: Schabracq MJ, Winnubst JAM, Cooper CL. The Handbook of Work and Health Psychology. Hoboken, New Jersyey: John Wiley & Sons; 2003; 2:383-426.

46. Schaufeli WB, Martinez IM, Pinto AM, Salanova M, Bakker AB. Burnout and engagement in university students: A cross-national study. J Cross Cult Psychol. 2002; 33: 464-481.

47. Maslach C, Schaufeli WB, Leiter MP. Job burnout. Annu Rev Psychol. 2001; 52: 397-422.

48. Jöreskog KG, Sörbom D. LISREL 8*:* Structural equation modeling with the SIMPLIS command language. Chicago, IL, US: Scientific Software International; Hillsdale, NJ, US: Lawrence Erlbaum Associates, Inc.; 1993.

49. Kline RB. Convergence of structural equation modeling and multilevel modeling. In: Williams M, Vogt WP. The SAGE handbook of innovation in social research methods. London: SAGE Publications Ltd; 2011: 562-589.

50. Dyrbye LN, West CP, Satele D, et al. Burnout among US medical students, residents, and early career physicians relative to the general US population. Acad Med. 2014; 89: 443-451.

For peer review only

For peer review only - http://bmjopen.bmj.com/site/about/guidelines.xhtml

51. Xanthopoulou D, Baker AB, Heuven E, Demerouti E, Schaufeli WB. Working in the sky: A diary study on work engagement among flight attendants. J Occup Health Psych. 2008; 13:345-356.

52. Lyubomirsky S, King L, Diener E. The benefits of frequent positive affect: Does happiness lead to success?. Psychol Bull. 2005; 131:803-855.

53. Avey JB, Reichard RJ, Luthans F, Mhatre KH. Meta-analysis of the impact of positive psychological capital on employee attitudes, behaviors, and performance. Hum Resour Dev Q. 2011; 22:127-152.

54. Avey JB, Luthans F, Jensen SM. Psychological capital: A positive resource for combating employee stress and turnover. Hum Resour Manage. 2009; 48:677-693.

55. Tims M, Bakker AB, Derks D. The impact of job crafting on job demands, job resources, and well-being. J Occup Health Psych. 2013; 18:230-240.

56. LePine JA, Podsakoff NP, LePine MA. A meta-analytic test of the challenge stressor-hindrance stressor framework: An explanation for inconsistent relationships among stressors and performance. Acad Manage J, 2005; 48:764-775.

57. Schaufeli WB, Bakker AB, Van der Heijden FM, Prins JT. Workaholism, burnout and well-being among junior doctors: The mediating role of role conflict. Work Stress. 2009; 23:155-172.

58. Conrad P. Learning to doctor: Reflections on recent accounts of the medical school years. J Health Soc Behav. 1988; 29:323-332.

59. Kashdan TB, Rottenberg J. Psychological flexibility as a fundamental aspect of health. Clin Psychol Rev. 2010; 30: 865-878.

60. Cohen S. Social relationships and health. Am Psychol. 2004; 59:676-684.

61. Berkman LF, Glass T, Brissette I, Seeman TE. From social integration to health: Durkheim in the new millennium. Soc Sci Med. 2000; 51:843-857.

For peer review only - http://bmjopen.bmj.com/site/about/guidelines.xhtml

62. Van der Doef M, Maes S. The job demand-control (-support) model and psychological well-being: a review of 20 years of empirical research. Work Stress. 1999; 13: 87-114.

63. Schaefer C, Coyne JC, Lazarus RS. The health-related functions of social support. J Behav Med. 1981; 4:381-406.

64. Zuckerman M. Attribution of success and failure revisited, or: The motivational bias is alive and well in attribution theory. J Pers. 1979; 47: 245-287.

65. Schaufeli WB, Taris TW, Van Rhenen W. Workaholism, burnout, and work engagement: Three of a kind or three different kinds of employee well-being?. Appl Psychol-Int Rev. 2008; 57:173-203.

66. Høigaard R, Giske R, Sundsli K. Newly qualified teachers' work engagement and teacher efficacy influences on job satisfaction, burnout, and the intention to quit. Eur J Teach Educ. 2012; 35:347-357.

67. Montgomery AJ, Panagopolou E, Benos A. Work–family interference as a mediator between job demands and job burnout among doctors. Stress Health. 2006; 22:203-212.

68. Leiter MP, Durup MJ. Work, home, and in-between: A longitudinal study of spillover. J Appl Behav Sci. 1996; 32:29-47.

69. Demerouti E, Bakker AB, Bulters AJ. The loss spiral of work pressure, work–home interference and exhaustion: Reciprocal relations in a three-wave study. J Vocat Behav. 2004; 64:131-149.

70. De Lange, A. H. (2005). What about causality?: examining longitudinal relations between work characteristics and mental health. Unpublished dissertation. Nijmegen, The Netherlands. Radboud University Press; 2005.

71. Podsakoff PM, MacKenzie SB, Lee JY, Podsakoff NP. Common method biases in behavioral research: A critical review of the literature and recommended remedies. J Appl Psychol. 2003; 88:879-903.

For peer review only - http://bmjopen.bmj.com/site/about/guidelines.xhtml

Figure Legends

Figure 1

Tested path model of job demands (workload, job insecurity, work-life conflict), job resources (autonomy, colleague support, supervisor support), personal resources (psychological capital, self-compassion, psychological flexibility), tenure, burnout, and work engagement in a Study on the Role of Personal Resources for Work Engagement and Burnout, 2017[d-f]

For peer review only

Figure 1

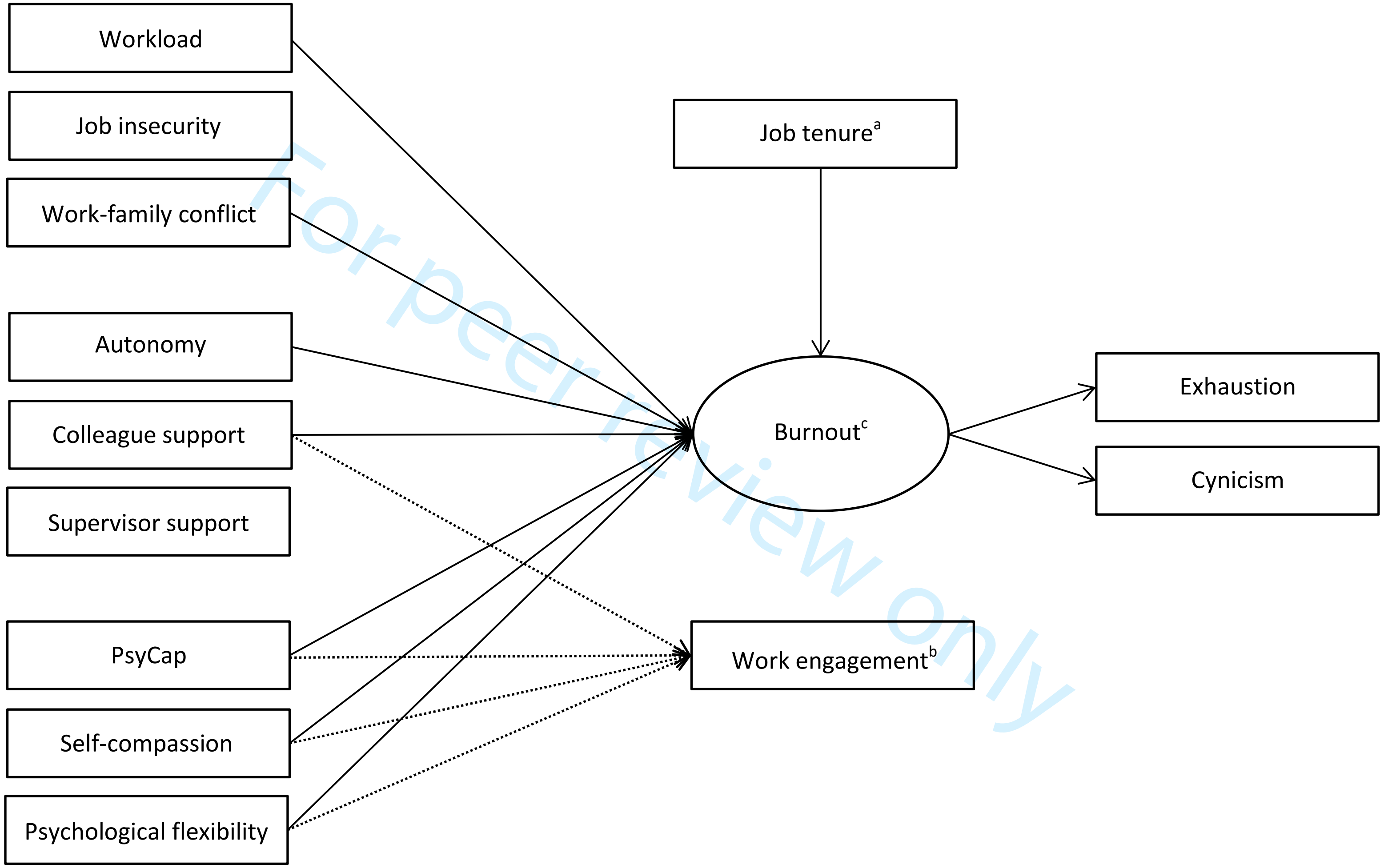

Abbreviation: PsyCap indicates psychological capital.
[a]Job tenure was measured with the item 'how long have you been working in your current job?'
[b]For ease of reading, paths between resources and work engagement are depicted by dotted lines instead of solid lines.

For peer review only

[c]Burnout as latent variable is based on the observed variables exhaustion and cynicism.
[d]The authors allowed covariation between all predictor variables, as well as between job tenure and autonomy. They also allowed covariation of error variance between exhaustion and cynicism with work engagement. For ease of reading, covariation is not depicted in the model.
[e]Testing the initial path model for specialists and residents separately revealed that only job tenure was related to the outcomes and was solely included as control variable.
[f]The final model showed a good fit to the data with= $X^2 = 132.33$ (75), $p < .001$, X^2/df = 1.76, CFI = .96, RMSEA = .05, SRMR = .06. The model explained 53.9 % of the variance in burnout, and 27.9 % of variance in work engagement.

For peer review only

Reporting checklist for cross sectional study.

Based on the STROBE cross sectional guidelines.

Instructions to authors

Complete this checklist by entering the page numbers from your manuscript where readers will find each of the items listed below.

Your article may not currently address all the items on the checklist. Please modify your text to include the missing information. If you are certain that an item does not apply, please write "n/a" and provide a short explanation.

Upload your completed checklist as an extra file when you submit to a journal.

In your methods section, say that you used the STROBE cross sectional reporting guidelines, and cite them as:

von Elm E, Altman DG, Egger M, Pocock SJ, Gotzsche PC, Vandenbroucke JP. The Strengthening the Reporting of Observational Studies in Epidemiology (STROBE) Statement: guidelines for reporting observational studies.

		Reporting Item	Page Number
Title	#1a	Indicate the study's design with a commonly used term in the title or the abstract	1
Abstract	#1b	Provide in the abstract an informative and balanced summary of what was done and what was found	2-3
Background / rationale	#2	Explain the scientific background and rationale for the investigation being reported	4-6
Objectives	#3	State specific objectives, including any prespecified hypotheses	7
Study design	#4	Present key elements of study design early in the paper	7-8
Setting	#5	Describe the setting, locations, and relevant dates, including periods of recruitment, exposure, follow-up, and data collection	7
Eligibility criteria	#6a	Give the eligibility criteria, and the sources and methods of selection of participants.	7
	#7	Clearly define all outcomes, exposures, predictors, potential confounders, and effect modifiers. Give diagnostic criteria, if applicable	8-11
Data sources / measurement	#8	For each variable of interest give sources of data and details of methods of assessment (measurement). Describe comparability of assessment methods if there is more than one group. Give information separately for for exposed and unexposed groups if applicable.	7

For peer review only

Bias	#9	Describe any efforts to address potential sources of bias	25
Study size	#10	Explain how the study size was arrived at	7
Quantitative variables	#11	Explain how quantitative variables were handled in the analyses. If applicable, describe which groupings were chosen, and why	11
Statistical methods	#12a	Describe all statistical methods, including those used to control for confounding	11-12
	#12b	Describe any methods used to examine subgroups and interactions	11
	#12c	Explain how missing data were addressed	See note 1
	#12d	If applicable, describe analytical methods taking account of sampling strategy	n/a
	#12e	Describe any sensitivity analyses	n/a
Participants	#13a	Report numbers of individuals at each stage of study—eg numbers potentially eligible, examined for eligibility, confirmed eligible, included in the study, completing follow-up, and analysed. Give information separately for for exposed and unexposed groups if applicable.	12
	#13b	Give reasons for non-participation at each stage	n/a
	#13c	Consider use of a flow diagram	n/a
Descriptive data	#14a	Give characteristics of study participants (eg demographic, clinical, social) and information on exposures and potential confounders. Give information separately for exposed and unexposed groups if applicable.	12-13
	#14b	Indicate number of participants with missing data for each variable of interest	n/a
Outcome data	#15	Report numbers of outcome events or summary measures. Give information separately for exposed and unexposed groups if applicable.	14, 16
Main results	#16a	Give unadjusted estimates and, if applicable, confounder-adjusted estimates and their precision (eg, 95% confidence interval). Make clear which confounders were adjusted for and why they were included	17-20
	#16b	Report category boundaries when continuous variables were categorized	n/a
	#16c	If relevant, consider translating estimates of relative risk into absolute risk for a meaningful time period	n/a
Other analyses	#17	Report other analyses done—e.g., analyses of subgroups and interactions, and sensitivity analyses	n/a

For peer review only

Key results	#18	Summarise key results with reference to study objectives	20-21
Limitations	#19	Discuss limitations of the study, taking into account sources of potential bias or imprecision. Discuss both direction and magnitude of any potential bias.	24-25
Interpretation	#20	Give a cautious overall interpretation considering objectives, limitations, multiplicity of analyses, results from similar studies, and other relevant evidence.	21-24
Generalisability	#21	Discuss the generalisability (external validity) of the study results	26
Funding	#22	Give the source of funding and the role of the funders for the present study and, if applicable, for the original study on which the present article is based	28

Author notes

1. 12 - incomplete cases were excluded from the analyses.

The STROBE checklist is distributed under the terms of the Creative Commons Attribution License CC-BY. This checklist was completed on 12. April 2019 using https://www.goodreports.org/, a tool made by the EQUATOR Network in collaboration with Penelope.ai

For peer review only

Keep the fire burning: a survey study on the role of personal resources for work engagement and burnout in medical residents and specialists in the Netherlands

Journal:	*BMJ Open*
Manuscript ID	bmjopen-2019-031053.R1
Article Type:	Original research
Date Submitted by the Author:	31-Jul-2019
Complete List of Authors:	Solms, Lara; University of Amsterdam, Work and Organizational Psychology; Erasmus Medical Center - Sophia Children's hospital, Pediatrics<br>van Vianen, Annelies; University of Amsterdam, Work and Organizational Psychology<br>Theeboom, Tim; Vrije Universiteit Amsterdam, School of Business and Economics<br>Koen, Jessie; University of Amsterdam, Work and Organizational Psychology<br>de Pagter, Anne; Erasmus Medical Center - Sophia Children's hospital<br>De Hoog, Matthijs; Erasmus Medical Centre - Sophia Childrens Hospital, Pediatrics/ Pediatric Intensive Care Unit
**Primary Subject Heading**:	Mental health
Secondary Subject Heading:	Medical education and training
Keywords:	Work engagement, Burnout, Personal resources, Professional development

SCHOLARONE™ Manuscripts

For peer review only - http://bmjopen.bmj.com/site/about/guidelines.xhtml

Keep the fire burning: a survey study on the role of personal resources for work engagement and burnout in medical residents and specialists in the Netherlands

Lara Solms, MSc, Annelies E.M. van Vianen, PhD, Tim Theeboom, PhD, Jessie Koen, PhD, Anne P.J. de Pagter, MD, PhD, Matthijs de Hoog, MD, PhD on behalf of the Challenge & Support Research Network

L. Solms is PhD candidate, Department of Work and Organizational Psychology, University of Amsterdam, Amsterdam, The Netherlands, PhD candidate, Department of Pediatrics, Erasmus MC-Sophia Children's Hospital, Rotterdam, The Netherlands

A.E.M van Vianen is professor of work and organizational psychology, Department of Work and Organizational Psychology, University of Amsterdam, Amsterdam, The Netherlands

T. Theeboom is assistant professor, School of Business and Economics, Vrije Universiteit Amsterdam, Amsterdam, The Netherlands

J. Koen is assistant professor, Department of Work and Organizational Psychology, University of Amsterdam, Amsterdam, The Netherlands

A.P.J. de Pagter* is pediatrician-hematologist, Department of Pediatrics, Erasmus MC-Sophia Children's Hospital, Rotterdam, The Netherlands and LUMC, Leiden, the Netherlands

M. de Hoog* is pediatric intensivist, professor of postgraduate medical education, director of pediatric residency training & director of PICU, Erasmus MC-Sophia Children's Hospital, Rotterdam, The Netherlands

* Anne P.J. de Pagter and Matthijs de Hoog contributed equally to this paper.

Correspondence should be addressed to Anne P.J. de Pagter, Department of Pediatrics, Erasmus MC-Sophia Children's Hospital, Rotterdam, Sp-2430 Wytemaweg 80, 3015 CN Rotterdam, The Netherlands; telephone: +31107032435; e-mail: p.depagter@erasmusmc.nl

For peer review only

Word count: 6559

Keywords: burnout, work engagement, personal resources, professional development

For peer review only

For peer review only - http://bmjopen.bmj.com/site/about/guidelines.xhtml

ABSTRACT

Objectives

The high prevalence of burnout among medical residents and specialists raises concerns about the stressful demands in healthcare. This study investigated which job demands and job- and personal resources are associated with work engagement and burnout, and whether the effects of these demands and resources differ for medical residents and specialists.

Design

In a survey study among residents and specialists, we assessed job demands, job resources, personal resources, work engagement and burnout symptoms using validated questionnaires (January to December 2017). Results were analyzed using multivariate generalized linear model, ordinary least squares regression analyses, and path analyses.

Setting

Six academic and general hospitals in the Netherlands.

Participants

A total number of 124 residents and 69 specialists participated in this study. Participants worked in the fields of pediatrics, internal medicine, and neurology.

Results

The associations of job and personal resources with burnout and work engagement differed for residents and specialists. Psychological capital was associated with burnout only for specialists ($b = -.58$, $p < .001$), whereas psychological flexibility was associated with burnout only for residents ($b = -.31$, $p < .001$). Colleague support ($b = .49$, $p < .001$) and self-compassion ($b = -.33$, $p = .004$) were associated with work engagement only for specialists.

Conclusions

This study suggests that particularly personal resources safeguard the work engagement and lessen the risk of burnout of residents and specialists. Both residents and specialists benefit

For peer review only - http://bmjopen.bmj.com/site/about/guidelines.xhtml

from psychological capital to maintain optimal functioning. In addition, residents benefit from psychological flexibility while specialists benefit from colleague support. Personal resources seem important protective factors for physicians' work engagement and well-being. When promoting physician well-being, a one-size-fits-all approach might not be effective but, instead, interventions should be tailored to the specific needs of specialists and residents.

Article summary

Strengths and limitations of this study

- The participation of both residents and specialists from different specialties and the high response rate (78%) allow for a realistic display of the demands and resources present during different career stages.
- This is the first study to show that the personal resources psychological capital, psychological flexibility, and self-compassion are important protective factors for residents and/or staff when fostering work engagement and combatting burnout.
- This study reveals that different demands and resources relate to burnout and work engagement for specialists and residents, pointing to unique problems that require unique solutions.
- All data are based on self-reports, which limits drawing strong conclusions about objectively existing job characteristics and their associations with burnout and work engagement.
- Although theory and research point to causal relationships between demands and burnout and resources and work engagement, the study is limited by the fact that its cross-sectional design does not infer causality.

For peer review only - http://bmjopen.bmj.com/site/about/guidelines.xhtml

INTRODUCTION

Healthcare professionals are well-known for their high work engagement, that is, their absorption by their work and dedication to patient care.[1-2] Unfortunately, such vigor and dedication can also have a flipside. Healthcare professionals work in a very complex system with long and irregular working hours and need to deal with various high job demands such as time pressure, emotionally taxing patient-interactions, work-family conflict and job insecurity. These high job demands cause alarming high rates of burnout symptoms such as emotional exhaustion and cynicism in healthcare compared to other professions.[3-7]Among physicians, medical residents seem to be especially at risk for burnout, with burnout symptoms often being reported by one out of four residents.[3, 8-10] This is hardly surprising: In addition to the job demands that all physicians face, residents also face high educational demands, need to get used to clinical rotations and shifts, experience high responsibility yet limited autonomy, and experience high uncertainty about their future career.[7,11] Additionally, residency marks a period that is characterized by stressful and demanding life events such as marriage and getting children.

Obviously, burnout has a strong negative impact on healthcare professionals and their employers. Most important, burnout symptoms are associated with decreased quality of care through delayed decision making, unprofessional work behaviors (i.e., conflict), and suboptimal patient care (i.e., not adequately discussing treatment options with patients or making medical errors).[7-9] Also, burnout is accompanied by long-term sickness leave and early retirement.[7] It is the highly critical work-environment of healthcare professionals and the high-stake consequences of their functioning that call for a deeper understanding of the demands and resources that relate to the work engagement and well-being (i.e., a lack of burnout) of these professionals. Similarly, it is important to understand which factors can

For peer review only - http://bmjopen.bmj.com/site/about/guidelines.xhtml

help physicians to stay motivated in spite of the demands they face. An important result of motivation is work engagement, a positive work-related state of mind characterized by vigor, dedication, and absorption.[1,12] Work engagement has been linked to employee performance across professions,[13-14] including healthcare.[15] Healthcare professionals who are engaged are also less likely to commit medical errors.[16] Consequently, both (a lack of) burnout and work engagement are important components for the optimal functioning of healthcare professionals.

Earlier research conceptualized burnout and work engagement as opposite poles of a continuum that are mutually exclusive.[17] Recent research[18] has evidenced that burnout and work engagement are (negatively) related, yet different constructs (i.e., low burnout does not necessarily imply high engagement). Furthermore, these studies have shown that job characteristics that are associated with the prevalence of burnout are different from those that are associated with work engagement. Surprisingly, few of these studies were carried out in health care settings. Yet, knowledge of the job characteristics that relate to burnout and work engagement among healthcare professionals is essential to develop tailored interventions (e.g., training, coaching) that support these professionals in their optimal functioning.[19]

Theoretical Framework: The Job Demands – Resources Model

The Job Demands - Resources (JD-R) model[12] proposes that work engagement and well-being are promoted when (healthcare) professionals have job resources that help them to cope with high job demands and that bolster their motivation. Generally, the JD-R model differentiates between two universal types of characteristics that people find in their jobs: that is job demands on the one hand and job resources on the other hand. Job demands refer to "those physical, psychological, social, or organizational aspects of the job that require sustained physical, and/or psychological effort and are therefore associated with

For peer review only - http://bmjopen.bmj.com/site/about/guidelines.xhtml

physiological and/or psychological costs”.[12] Examples of job demands are workload, time pressure, and emotional demands.[12] High job demands require professionals to spend sustained effort in order to meet perceived demands, which gradually drains resources and ultimately leads to depletion and exhaustion.[20] That being said, job demands are considered the prime factor leading to burnout.[12] Fortunately, professionals also have job resources that support them in coping with job demands. Such job resources, i.e., “physical, psychological, social, or organizational aspects of the job that help to either achieve work goals, reduce job demands and the associated physiological and psychological costs, or stimulate personal growth, learning, and development”[20] can comprise both situational/external and personal/internal resources.[21-22] Situational resources are, for example, colleague and supervisory support, and the amount of autonomy professionals have in their work.[18,23] However, resources are not exclusively found in the environment, but people can also create them for themselves.[24] Personal resources refer to individual psychological states such as an individual’s psychological capital (including self-efficacy, hope, optimism and resilience), self-compassion (treating oneself with kindness when things go wrong), and cognitive flexibility (the ability to choose behaviors that are in line with ones goals and values) that mirror people’s perception to control and impact successfully upon the environment.[25-26]

Apart from supporting employees in coping with job demands, situational and personal resources are also important in their own right as they are considered the prime factor leading to work engagement.[27] Meta-analytic studies have shown, for example, that colleague and supervisory support[13] and optimism and self-efficacy[28] were positively related to work engagement.

The current study builds on the JD-R model that considers burnout and work engagement as independent yet correlated constructs and job demands and (situational and personal) resources as the main predictors of burnout and work engagement, respectively. Although the

For peer review only - http://bmjopen.bmj.com/site/about/guidelines.xhtml

relationships between demands and resources as antecedents and burnout and work engagement as outcomes are confirmed in numerous studies, research also shows that the strength of these relationships varies. This variation is likely due to the different professional samples and work contexts that were studied.[29-31] While residents and specialists work in the same occupational setting, they may face different job characteristics and, hence, different job demands and resources. As such, it is particularly relevant to examine whether the job demands, resources and outcomes, and the relationships among these variables, differ between residents and specialists. Insight into this topic can inform stakeholders how to regulate workplace practices in order to foster physician well-being and work engagement. Importantly, such information can ensure the effectiveness of interventions, because it allows us to tailor interventions to a specific situation or group and tap into personal and situational characteristics that can be changed at the individual and the group level, respectively. In the current study, we therefore investigate how job demands (workload, job insecurity, work-family conflict), situational resources (autonomy, supervisor support, colleague support) and personal resources (psychological capital, self-compassion, psychological flexibility) relate to work engagement and burnout among specialists and residents.

METHOD

Study population

From January to December 2017, we collected data from attending (specialists) and resident physicians at four academic and two general hospitals in the Netherlands. The physicians were specialized or trained in pediatrics, internal medicine, or neurology. Because this study is part of a larger program offering individual coaching to physicians, the sample consists of physicians that signed up for the coaching program or control participants. The choice of departments and hospitals that were invited for participation in this study was based on internal logistics. That is, because the coaching program was only offered to physicians and

For peer review only - http://bmjopen.bmj.com/site/about/guidelines.xhtml

residents from the pediatrics department, a relatively high number of participants in this sample are pediatricians. A comparison of the gender demographics of this sample with the broader population indicate that the sample of residents is representative of the hospital population. However, female specialists are overrepresented in our sample. Limitations of generalizability will be discussed.

Procedure

All physicians were invited by email to complete an online survey. Participation was voluntary; participants provided informed consent for participation in the study. We took measures to safeguard the anonymity and confidentiality of all participants and ethical approval was obtained by the ethical board of the University of Amsterdam.

Measures

To capture the different components of the JD-R model, we included, job demands, job resources, personal resources, burnout and work engagement in the survey, as well as demographics.

Job demands. Job demands were assessed with three scales: workload, job insecurity, and work family conflict. *Workload* was assessed with four items from the Quantitative Workload Inventory[32] and two additional items (α = .85). An example item measuring quantitative workload is: "How often does your job require you to work fast?" The two additional items were "How often does your job require you to work overtime? "and "How often do you experience emotional strain from your job?". The items were scored on a five-point scale ranging from 1 ("never") to 5 ("always"). Higher scores indicate higher frequency, i.e., higher workload.

Job insecurity, that is, "the perceived threat of job loss and the worries related to that threat"[33] was measured with an adapted version of the Job Insecurity Scale.[34] The scale consisted of five items (α = .83) including "Chances are, that in the future I won't be able to find the job

For peer review only - http://bmjopen.bmj.com/site/about/guidelines.xhtml

that I want" or "I am feeling insecure about the future of my career". The items were scored on a seven-point scale ranging from 1 ("not at all applicable") to 7 ("very applicable"). Higher scores indicate stronger applicability, i.e., higher job insecurity.

Work-Family Conflict was measured with four items of the Work-Family Conflict Scale (α = .87) measuring the extent to which "the general demands of, time devoted to, and strain created by the work interfere with performing family-related responsibilities".[35] An example item is: "The demands of my work interfere with my home and family life". The items were scored on a seven-point scale ranging from 1 ("not at all applicable") to 7 ("very applicable"). Higher scores indicate stronger agreement with the proposition, i.e., higher work-family conflict.

Job resources. Job resources encompassed autonomy, supervisor support, and colleague support.

Autonomy was measured with nine items from the Work Design Questionnaire[36] (α = .93) assessing perceived autonomy with regard to work scheduling and methods and decision-making. Example items include "The job allows me to plan how I do my work", "The job provides me with significant autonomy in making decisions" and "The job allows me to make decisions about what methods I use to complete my work" respectively. The items were scored on a seven-point scale ranging from 1 ("totally disagree") to 7 ("totally agree"). Higher scores indicate stronger agreement with the proposition, i.e., higher autonomy.

Supervisor support, that is, the experienced psychological and work support from the supervisor was assessed with six items from Vinokur, Schul, and Caplan[37] (α = .95). Example items include "My supervisor provides me with encouragement" or "My supervisor says things that raise my self-confidence". For residents, supervisory support measured the support received from the training supervisor, whereas for specialists, supervisory support measured the support received from the head of the department. The items were scored on a

For peer review only - http://bmjopen.bmj.com/site/about/guidelines.xhtml

seven-point scale ranging from 1 (“totally disagree”) to 7 (“totally agree”). Higher scores indicate stronger agreement with the proposition, i.e., higher supervisor support.

Colleague support, the experienced psychological and work support from colleagues, was assessed with the same six items as supervisor support (α = .94), but the items referred to colleagues instead of the supervisor. Also here, higher scores indicate stronger agreement with the proposition, i.e., higher colleague support.

Personal resources. We included three personal resources: psychological capital, self-compassion, and psychological flexibility.

Psychological capital was measured with 12 items reflecting hope, optimism, resilience, and efficacy from the validated Dutch version of the Psychological Capital Questionnaire[38-39] (α = .88). The items include “Right now I see myself as being pretty successful at work” (hope), “I always look on the bright side of things regarding my job” (optimism), “When I have a setback at work, I have trouble recovering from it, moving on (R)” (resilience) and “When encountering difficult problems in my work, I know how to solve them” (efficacy). The items were scored on a seven-point scale ranging from 1 (“totally disagree”) to 7 (“totally agree”). Higher scores indicate stronger agreement with the proposition, i.e., higher psychological capital.

Self-compassion, entailing “treating oneself with kindness, recognizing one’s shared humanity, and being mindful when considering negative aspects of oneself”[40] was measured with six items from the Self-Compassion Scale[40] (α = .72). Example items are: “When I am going through a very hard time, I give myself the caring and tenderness I need” (self-kindness), “I try to see my failings as part of the human condition” (common humanity), and “When something painful happens I try to take a balanced view of the situation” (mindfulness). The items were scored on a five-point scale ranging from 1 (“rarely”) to 5 (“almost always”). Higher scores indicate higher frequency, i.e., higher self-compassion.

For peer review only - http://bmjopen.bmj.com/site/about/guidelines.xhtml

Psychological flexibility, that is, the ability to flexibly take appropriate action towards achieving goals and values, even in the presence of challenging or unwanted events[41] was measured with seven items of the Work Acceptance and Action Questionnaire[42] (α = .81). Example items include "I am able to work effectively in spite of any personal worries that I have" and "I can work effectively, even when I doubt myself." The items were scored on a five-point scale ranging from 1 ("rarely") to 5 ("almost always"). Higher scores indicate higher frequency, i.e., higher psychological flexibility.

Outcomes. The outcome variables included in this study were burnout symptoms and work engagement.

Burnout was measured with the Dutch version[43] of the Maslach Burnout Inventory – General Survey[44]. The instrument consists of three subscales measuring exhaustion, cynicism and professional efficacy. Because exhaustion and cynicism constitute the essence of the burnout syndrome[45], we only measured these two components. *Exhaustion* was measured with 5 items (α = .84). An example item is: "Working all day is really a strain for me." The items were scored on a seven-point scale ranging from 1 ("totally disagree") to 7 ("totally agree"). *Cynicism* was measured with 4 items (α = .77). An example item is: "I noticed that I have got too much distance from my work." The items were scored on a seven-point scale ranging from 1 ("totally disagree") to 7 ("totally agree"). Higher scores indicate stronger agreement with the proposition, i.e., higher exhaustion and cynicism, respectively.

Work engagement, including vigor, dedication, and absorption at work was measured with nine items from the Utrecht Work Engagement Scale[46] (α = .90). Example items include: "When I get up in the morning, I feel like going to work" (vigor), "I am enthusiastic about my job" (dedication), and "When I am working I forget everything around me" (absorption). The items were scored on a seven-point scale ranging from 1 ("never") to 7 ("always"). Higher scores indicate higher frequency, i.e., higher work engagement.

For peer review only - http://bmjopen.bmj.com/site/about/guidelines.xhtml

Statistical analysis

Factor structure. To examine whether the items loaded on their respective scales, we performed separate confirmatory factor analyses (CFAs) for the scales representing job demands, job resources, and personal resources, respectively. As each of these predictors consists of three scales, we compared a three-factor model to a one-factor model. We report the factor loadings and the commonly used model fit criteria, that is, the chi-square goodness-of-fit value, the chi-square divided by the degrees of freedom (CMIN/DF), the comparative fit index (CFI), the root mean square error of approximation (RMSEA) and the standardized root mean squared residual (SRMR).

Between group variance. Because participants can be considered as nested within (four) hospitals and (three) specializations, we first assessed between group variance within our data. A multilevel mixed-method analysis estimating a random intercept model was conducted to calculate between level-2 variance[a].

Control variables. We explored the association between potential control variables (i.e., age, gender, having children, job tenure, signed up for coaching) and the dependent variables by means of regression analyses for residents and specialists separately.

Path analysis. The relationships between the independent (job demands, job resources, and personal resources) and dependent variables (exhaustion, cynicism, and work engagement), were examined with path analysis using IBM SPSS AMOS 25 (IBM SPSS, Chicago). In a first step, we modeled a latent variable termed burnout based on the observed variables exhaustion and cynicism. Modeling these two outcome variables on one latent variable was justified both theoretically[45,47] and statistically (correlation of $r = .58$, $p < .01$ between exhaustion and cynicism).

[b] The final Hessian matrix was not positive definite as the intercept variance was zero.

For peer review only - http://bmjopen.bmj.com/site/about/guidelines.xhtml

A path model with independent variables and work engagement and burnout as dependent variables was tested using a covariance matrix as input and maximum likelihood estimation. This analysis adequately captures the nature of the associations between the independent and dependent variables and was therefore chosen over regular OLS regression analyses. Furthermore, this analysis allowed for a multi-group comparison, testing possible differences in model estimates between residents and specialists. Again, we report the commonly used model fit criteria as described earlier.

Patient and public involvement

This study investigated factors associated with work engagement and burnout in medical specialists and residents. No patients or public representatives were involved in the study.

RESULTS

In total, we invited a number of 247 physicians to take part in this survey of whom 75 physicians had signed up for a personal coaching program that would start in a few months. A total number of *N* = 193 physicians were included in this study after application of inclusion criteria[b] (response rate = 78 %). The study population included 151 females (78.2 %) and 42 males (21.8 %). The mean age was 36.5 years (*SD* = 8.5). 124 residents (64.2 %) and 69 medical specialists (35.8 %) participated. Participants were working in the field of pediatrics (*n* = 142; 73.6 %), neurology (*n* = 14; 7.3 %), and internal medicine (*n* = 37; 19.2 %). See table 1 for a description of participants' characteristics. Internal consistency (Cronbach's alpha) was acceptable for all scales (see Table 2).

[b] Inclusion criteria: minimal response time >15 minutes, survey was filled out no later than one week after the first coaching session; survey progress $\geq$ 80 percent

For peer review only - http://bmjopen.bmj.com/site/about/guidelines.xhtml

Table 1

Demographics of Residents and Specialists Participating in a Study on the Role of Personal Resources for Work Engagement and Burnout, 2017[a]

Characteristics	Residents	Specialists
	No. (% of 124)	No. (% of 69)
**Gender**		
Female	101 (81.5%)	50 (72.5%)
Male	23 (18.5%)	19 (27.5)
**Age[b]**		
20 - 30 years	42 (33.9%)	1 (1.4%)
31 - 40 years	81 (65.3%)	28 (40.6%)
41 - 50 years	1 (0.8%)	22 (31.9%)
51 - 60 years	-	15 (21.7%)
61 years and older	-	3 (4.3%)
**Specialty**		
Pediatrics	87 (70.2%)	55 (79.7%)
Internal medicine	27 (21.8%)	10 (14.5%)
Neurology	10 (8.1%)	4 (5.8%)
**Signed up for coaching[c]**		
Yes	53 (42.7%)	36 (52.2%)
No	71 (57.3%)	33 (47.8%)
**Home situation**		
Children, one or more	51 (41.1%)	48 (69.6%)
No children	73 (58.9%)	21 (30.4%)

[a]This study was conducted at 4 academic and 2 general hospitals in The Netherlands. In this study, the authors investigated associations between job demands (workload, work-family conflict, job insecurity), job resources (autonomy, supervisor support, colleague support), personal resources (psychological capita, self-compassion, psychological flexibility) with work engagement and burnout.
[b]The residents taking part in this study were on average 31.9 years old, (standard deviation [SD] = 3.0); the specialists were on average 44.9 years old (SD = 8.7).
[c]Participants were registered to participate in an institutional coaching program or private coaching.

For peer review only

For peer review only - http://bmjopen.bmj.com/site/about/guidelines.xhtml

Table 2

Correlations between the Study Variables for Residents and Specialists and Internal Consistencies[c] of Study Variables in a Study on the Role of Personal Resources for Work Engagement and Burnout, 2017[a-c]

Study Variables	1	2	3	4	5	6	7	8	9	10	11	12	13	14
1. Signed up for coaching[d]	-	.11	.31*	-.27*	-.40**	.18	.02	.32**	.28*	.23	.16	-.26*	-.11	.05
2. Job tenure	.13	-	.02	-.22	-.10	.14	-.05	.11	.19	.21†	.00	.12	.10	.02
3. Workload	-.09	.08	**0.85**	.18	.46**	-.14	-.00	-.16	-.14	-.16	.06	.33**	.27*	.02
4. Job insecurity	-.16†	.18*	.23*	**0.83**	.10	-.29*	-.27*	-.21†	-.53**	-.42**	-.29*	.29*	.38**	-.41**
5. Work-family conflict	-.13	.11	.36**	.15	**0.87**	-.32**	-.16	-.10	-.25*	-.36**	.01	.42**	.27**	-.07
6. Autonomy	.13	.13	-.29**	-.18*	-.11	**0.93**	.21	.29*	.55**	.37**	.18	-.35**	-.28*	.38**
7. Colleague support	.08	-.03	-.19*	-.24**	-.06	.18*	**0.94**	.08	.43**	.47**	.16	-.47**	-.58**	.60**
8. Supervisor support	-.07	-.17	.01	-.07	.14	.20*	.08	**0.95**	.41**	.25*	.31**	-.15	-.16	.25*
9. PsyCap	.13	-.13	-.22*	-.38**	-.21*	.33**	.26**	.19*	**0.88**	.68**	.37**	-.50**	-.60**	.58**
10. Self-compassion	.13	-.13	-.32**	-.41**	-.33**	.15	.22*	.09	.51*	**0.72**	.25*	-.33**	-.50**	.32**
11. Psych. flexibility	.06	.03	-.01	-.13	-.02	.30**	.26**	.05	.37*	.13	**0.81**	.10	-.17	.32**
12. Exhaustion	-.05	.15†	.35**	.22*	.51**	-.04	-.23*	-.07	-.32*	-.45**	-.26**	**0.84**	.67**	-.50**
13. Cynicism	.14	.26**	.23**	.23*	.23*	-.21*	-.33**	-.17	-.42**	-.26**	-.37**	.58**	**0.77**	-.67**
14. Work engagement	-.05	-.04	-.12	-.15	-.14	.31**	.24**	.20*	.54**	.29**	.38**	-.40**	-.62**	**0.90**

Abbreviations: PsyCap indicates psychological capital; psych. flexibility indicates psychological flexibility.
[a]The values below the diagonal refer to residents. The values above the diagonal refer to specialists.
[b]Statistical significance of correlation coefficients is indicated by the following symbols: †p < .10. *p < .05. **p < .01.
[c]Internal consistencies (Cronbach alpha) are displayed on the diagonal in bold numbers.
[d]Participation (yes) was indicated with the number 1, participation (no) was indicated with the number 2.

For peer review only - http://bmjopen.bmj.com/site/about/guidelines.xhtml

Factor structure

All items loaded on their respective scales. Factor loadings were on average .73, .81, and .60 for job demands, job resources, and personal resources, with three items loading below .40 and a minimal loading of .28. These items were included because the scales were validated test instruments with overall high internal consistencies. The modification indices provided by the CFAs indicated that some items shared error variance. In order to improve the model fit, we allowed covariation of error variance between these items. Covariation was only allowed for items originating from the same scale. All three models provided adequate fit to the data with $X^2(86) = 153.74$, $p < .001$, $X^2/\text{df} = 1.79$, CFI = .95, RMSEA = .06, SRMR = .06; $X^2(180) = 312.75$, $p < .001$, $X^2/\text{df} = 1.74$, CFI = .97, RMSEA = .06, SRMR = .06, and $X^2(262) = 435.96$, $p < .001$, $X^2/\text{df} = 1.66$, CFI = .90, RMSEA = .06, SRMR = .07 for the three-factor models representing job demands, job resources and personal resources, respectively. Our results showed that the hypothesized three-factor model of these predictors provided a better fit to the data than a common factor model (e.g., fit indices of the common factor model of job demands: $X^2(89) = 864.96$, $p < .001$, $X^2/\text{df} = 9.72$, CFI = .46, RMSEA = .21, SRMR = .19). The differences in the chi-square goodness-of-fit value between the three-factor and the common factor models were significant, all *p's* < .001. These results allow us to conclude that the factor structure assumed in our path model is appropriate.

Between group variance

A multivariate GLM analysis confirmed that hospitals and specializations did not significantly differ on exhaustion, cynicism, and work engagement. Therefore, it was not necessary to account for group level effects when estimating the relationships between the independent and dependent variables.

Control variables

The results showed that only the control variables job tenure; $b = .23$, $p = .02$ (related to

For peer review only - http://bmjopen.bmj.com/site/about/guidelines.xhtml

exhaustion for residents), $b = .24$, $p = .02$ (related to cynicism for residents) and signed up for coaching[c]; $b = -.31$, $p = .01$ (related to exhaustion for specialists) were related to exhaustion, cynicism, or engagement. Therefore, and to save power, we only included job tenure and signed up for coaching as control variables in the further analyses.

Descriptives and group differences

Table 3 describes means, standard deviations and differences in study variables for residents and specialists, respectively. Independent sample t-tests were performed to investigate mean-level differences in study variables comparing residents and specialists. Compared to specialists, residents reported significantly lower workload ($M = 3.29$, $SD =.68$ vs. $M = 3.52$ $SD = .83$, $p^d < .05$), lower autonomy ($M = 4.10$, $SD =.99$ vs. $M = 5.19$, $SD = 1.02$, $p < .01$), and lower work engagement ($M = 4.93$, $SD = .77$ vs. $M = 5.21$ $SD = .88$, $p < .05$). However, residents reported significantly higher job insecurity than specialists ($M = 4.26$, $SD = 1.17$ vs. $M = 3.03$, $SD = 1.27$, $p < .01$).

[c] Response options were: 1 = yes, 2 = no
[d] Significance values were $p = .039$ when equal variances were assumed, and $p = .052$ when equal variances were not assumed.

For peer review only - http://bmjopen.bmj.com/site/about/guidelines.xhtml

Table 3

Means and Standard Deviations (SD) of Study Variables for Residents and Specialists in a Study on the Role of Personal Resources for Work Engagement and Burnout, 2017[c]

Study Variables	Residents[a]	Specialists[b]
	Mean (SD)	Mean (SD)
**Work demands**		
Workload*[d]	3.29 (.68)	3.52 (.83)
Job insecurity**	4.26 (1.17)	3.03 (1.27)
Work-family conflict	4.47 (1.14)	4.56 (1.32)
**Job resources**		
Autonomy**	4.10 (.99)	5.19 (1.02)
Colleague support	5.37 (.85)	5.38 (1.17)
Supervisor support	4.66 (1.39)	4.84 (1.49)
**Personal resources**		
PsyCap[†]	4.91 (.72)	5.11 (.75)
Self-compassion[†]	3.19 (.63)	3.36 (.65)
Psych. flexibility	3.53 (.61)	3.62 (.62)
**Outcomes**		
Exhaustion	2.55 (1.02)	2.40 (1.18)
Cynicism	2.24 (.99)	2.01 (1.07)
Work engagement*	4.93 (.77)	5.21 (.88)

Abbreviation: SD indicates standard deviation; PsyCap indicates psychological capital; psych. flexibility indicates psychological flexibility.
[a]A total number of 124 residents participated.
[b]A total number of 69 specialists participated.
[c]Differences in means between residents and specialists are indicated by the following significance values: [†]$p < .10$; *$p < .05$; **$p < .01$.
[d]Significance values were $p = .039$ with equal variances assumed, and $p = .052$ with equal variances not assumed.

For peer review only

Path analyses

Preliminary analyses. As suggested by Joreskög and Sörbom[48], we first specified an initial model based on our research question, and then adjusted the model according to the modification indices it produced, allowing covariation between all predictor variables, as well as covariation between job tenure and autonomy. Because both indicators of burnout highly correlated with engagement, we allowed covariation of error variance between exhaustion and cynicism with engagement. Testing the initial path model for specialists and residents separately revealed that the control variable signed up for coaching was not related to any of the two outcomes in both sub-samples. We, therefore, removed this variable from the analysis and continued the analysis with only job tenure as control variable.

Model fit. The path analysis showed a satisfactory fit to the data, $X^2(51) = 109.25$, $p < .001$, $X^2/\text{df} = 2.14$, CFI = .96, RMSEA = .06, SRMR = .06. In order to improve the model fit, we removed the paths that were non-significant for both residents and specialists.[49]

We removed the paths from job insecurity and supervisor support to burnout. Further, we removed the paths from workload, job insecurity, work-family conflict, autonomy, supervisor support and job tenure to work engagement as they were not significant, partly despite significant zero-order correlations between these variables (see Table 2). The model resulted in an improved fit of $X^2(75) = 132.33$, $p < .001$, $X^2/\text{df} = 1.76$, CFI = .96, RMSEA = .05, SRMR = .06. The tested model is presented in Figure 1. The model explained 53.9 % of the variance in burnout, and 27.9 % of variance in work engagement.

Relationships with burnout. The standardized path coefficients with burnout as outcome are presented in Figure 1.

Job demands. Separate tests for residents and specialists suggested that there were no differences between both groups: workload was positively related to burnout for residents (*b*

For peer review only - http://bmjopen.bmj.com/site/about/guidelines.xhtml

= .20, p = .011) and specialists (b = .22, p = .009). A multi-group comparison test confirmed that these relationships did not significantly differ, $p > .05$. Furthermore, separate tests for both groups suggested that work-family conflict as a job demand differed between residents and specialists: it was positively related to burnout for residents (b = .33, $p < .001$) but not for specialists ($p > .05$). However, these relationships did not significantly differ in a multi-group comparison test, $p > .05$.

Job resources. Separate tests for both groups suggested that there were differences between residents and specialists regarding autonomy: autonomy was positively related to burnout for residents (b = .19, p = .016), but not for specialists ($p > .05$). However, these differences did not significantly differ in a multi-group comparison test, $p > .05$. Furthermore, separate tests for both groups suggested that colleague support differed between residents and specialists: it was not related to burnout for residents ($p > .05$), but was negatively related to burnout for specialists (b = -.41, $p < .001$). However, these relationships did not significantly differ in a multi-group comparison test, p = .088.

Personal resources. Separate tests for both groups suggested that there were differences between residents and specialists regarding the personal resource psychological capital: psychological capital was not related to burnout for residents ($p > .05$), but was negatively related to burnout for specialists (b = -.58, $p < .001$). A multi-group comparison test confirmed that these relationships significantly differed, p = .003. Furthermore, separate tests for both groups suggested that self-compassion differed between residents and specialists: self-compassion was negatively related to burnout for residents (b = -.22, p = .017) but not related to burnout for specialists, $p > .05$. However, these relationships did not significantly differ in a multi-group comparison test, p = .072. Also, separate tests suggested that there were differences between residents and specialists regarding the personal resource psychological flexibility: it was related to burnout for residents (b = -.31, $p < .001$), but not

For peer review only - http://bmjopen.bmj.com/site/about/guidelines.xhtml

related to burnout for specialists ($p > .05$). A multi-group comparison test confirmed that these relationships did significantly differ, $p = .003$.

Relationships with work engagement. The standardized path coefficients with work engagement as outcome are presented in Figure 1.

Job resources. Separate tests suggested that there were differences regarding colleague support between residents and specialists: colleague support was not related to work engagement for residents ($p > .05$), but was positively related to work engagement for specialists ($b = .49$, $p < .001$). A multi-group comparison test confirmed that these relationships did significantly differ, $p = .001$.

Personal resources. Separate tests for both groups suggested that there were no differences between residents and specialists regarding psychological capital: psychological capital was positively related to work engagement for both residents ($b = .37$, $p < .001$) and specialists ($b = .55$, $p < .001$). A multi-group comparison test confirmed that these relationships did not significantly differ, $p > .05$. Furthermore, separate tests for both groups suggested that there were differences between residents and specialists regarding self-compassion: self-compassion was not related to work engagement for residents ($p > .05$), but was negatively related to work engagement for specialists ($b = -.33$, $p = .004$). A multi-group comparison test confirmed that these relationships did significantly differ, $p =.003$. Also, separate tests for both groups suggested that there were differences between residents and specialists regarding psychological flexibility: psychological flexibility was positively related to work engagement for residents ($b = .17$, $p = .035$), but not for specialists ($p > .05$). However, a multi-group comparison test showed that these relationships did not significantly differ, $p = .778$.

DISCUSSION

Main findings

For peer review only - http://bmjopen.bmj.com/site/about/guidelines.xhtml

The goal of this study was to gain insight on the prevailing demands and resources that contribute to burnout and work engagement in medical residents and specialists. This study revealed that residents and specialists face different demands in their work that cannot be measured by the same yardstick but instead require tailored solutions. Confirming prior studies on the stressful demands during residency[7], our data showed that residents as compared to specialists experienced less autonomy and felt more uncertain about the future of their job. Furthermore, symptoms of exhaustion and cynicism among residents increased with tenure, which may at least partly relate to growing feelings of job insecurity (see Table 2). Contrary to what has been reported in previous studies,[50] residents did not report significantly higher exhaustion and cynicism than specialists but, on average, felt less engaged with their work.

We suggest that specialists and residents resort to different resources to cope with their job demands. Although both groups have the same level of resources at their disposal, only certain resources contribute to the well-being of specialists and residents, respectively. While specialists benefit from psychological capital and colleague support, residents benefit especially from psychological flexibility and self-compassion. It is likely that residents and specialists - as a function of their role and the career phase they are in - use those resources that bring the greatest benefit when facing job demands at work. This will be discussed subsequently. In addition, psychological capital was found to play a role for the work engagement of both specialists and residents, which corroborates earlier findings among other professional groups.[51]

Physicians are exposed to high job demands, both during attendance and residency, which could harm their well-being.[12] However, our study suggests that job demands other than workload (e.g. job insecurity, work-family conflict) and a lack of resources (e.g., self-compassion, psychological capital, and psychological flexibility) play a prominent role in the

For peer review only - http://bmjopen.bmj.com/site/about/guidelines.xhtml

onset of burnout. The fact that the residents in this study reported a relatively lower workload than specialists, yet reported similar symptoms of burnout, underlines this notion. Generally, our findings suggest that for preventing burnout it is important to focus on those demands and resources that are most relevant for specific groups of physicians (e.g., specialists or residents). Specialists may particularly benefit from interventions that raise their psychological capital and – at the team level – foster team cohesion and support whereas residents may benefit relatively more from interventions that increase their self-compassion and flexibility.

Resources that buffer burnout among specialists and residents

Personal resources. Consistent with prior research,[52-53] psychological capital played an eminent role for the well-being of specialists. Psychological capital may reduce the risk of burnout in two ways. First, it can counteract the distress associated with a demanding workplace by regulating negative emotions.[54] Second, individuals with high psychological capital tend to perceive job demands as challenges rather than hindrances. That is, they associate job demands with personal gain or growth,[55] evoking positive emotions, instead of fear and threat, evoking negative emotions.[55-56] Given the buffering capacity of psychological capital,[52-53] it is surprising that we did not find the same result among residents. Instead, we found that flexibility and self-compassion rather than psychological capital contributed to the well-being of residents.

The importance of flexibility and self-compassion among residents may be due to their specific career phase, which is characterized by insecurity, constant feedback, and criticism. That is, residency is an extremely challenging period, in which residents have to deal with their newly gained responsibilities (i.e., managing uncertainty, breaking bad news) whilst also processing new information and continuously adapting to new organizational structures and teams. Residents have to shift regularly between their roles as trainee and doctor,[57]

For peer review only - http://bmjopen.bmj.com/site/about/guidelines.xhtml

experience high challenges at work, and are confronted with their relative lack of knowledge and skills when entering residency. More so, residents are taught from medical school on to be critical towards themselves, a necessity that is demanded in a high-stake work environment where carelessness can have radical consequences. Consequently, medical professionals likely adopt a rather self-critical attitude.[58] One way to deal with these stressful work events is to accept one's inexperience, forgive one's deficiencies (i.e., self-compassion) and remain effective despite self-doubt and worries (i.e., psychological flexibility). More specifically, residents need to internalize that despite the current healthcare culture, not-knowing, insecurities and mistakes are part of the journey and not a sign of weakness or failure. In addition, psychological flexibility may allow residents to shift between tasks and professional roles, as it facilitates adapting to fluctuating situational demands, shifting perspective, and reconfiguring mental resources.[59] Thus, being kind towards oneself and viewing one's own shortcomings as human can help to safeguard residents against the stressors uniquely present in residency.

Colleague support. In addition to psychological capital, colleague support also seems to promote the well-being of specialists. Numerous studies have indeed shown that social support is associated with both psychological and physical health outcomes[60-61] as it ameliorates the impact of stress and strain on health.[23,61-62] First, social support may involve emotional support, the feeling that one is loved and cared for.[63] Secondly, it may involve the provision and sharing of information.[63] Specialists work in relatively permanent teams with interdependent work relationships. Knowing their colleagues and their expertise well, they can ask for and receive emotional but also informational support. Colleagues can provide intimacy or reassurance during emotional and stressful events and they can assist in times of uncertainty and difficult medical inquiries that require another expert opinion.

For peer review only - http://bmjopen.bmj.com/site/about/guidelines.xhtml

Although our data does not allow any insights in the quality of support received from colleagues, it is possible that the quality of support is different for residents and specialists. Because of the nature of residency (e.g., competition and regular rotations) it is likely that colleague relationships are relatively less permanent and fruitful for residents. Although valuing their opinion, residents might not be convinced that they can ultimately lean on a fellow residents' opinion in solving medical problems. This could explain why residents benefit relatively less from colleague support than specialists.

Resources that foster work engagement among specialists and residents

Personal resources. Our finding that psychological capital is a personal resource that is vital for the work engagement of both residents and specialists corroborates with prior studies among other professional groups.[54-55]

Unexpectedly, self-compassion was negatively rather than positively associated with the work engagement of specialists, while we found no such effect for residents. It is possible that high levels of self-compassion represent a self-protective bias, which serves to deny responsibility for failure.[64] That is, high self-compassion may lean towards attributing failure to external factors (e.g., situational constrains, lack of help from others) allowing specialists to maintain positive perceptions of their capabilities. Over a longer period of time, this way of thinking about their own shortcomings may hamper specialists' personal development and work efforts, which may ultimately cause a reduction in work engagement. It is therefore worth exploring whether the benefits of self-compassion depend on time, or whether there is an optimal level - or perhaps a tipping point - at which self-compassion contributes to one's well-being.

Colleague support. Colleague support not only buffered the occurrence of burnout, but also fostered work engagement among specialists rather than residents. As argued above, specialists as opposed to residents work in more permanent teams. It has been consistently

For peer review only - http://bmjopen.bmj.com/site/about/guidelines.xhtml

found that the social support in these teams facilitates the work engagement of team members.[65]

Study strengths and limitations

The participation of both residents and specialists from different specialties and the high response rate (78 %) allowed for a realistic display of the demands and resources that physicians encounter during different stages of their career. Our research shows how different demands and resources relate to burnout and work engagement among specific groups of physicians, and, as such, advances our understanding of how to intervene when well-being or work engagement are at risk. This is an important first step in the prevention of burnout and the conservation of work engagement among healthcare professionals. Yet, our results also indicate that burnout and work engagement are highly interweaved, just as in numerous prior studies. [65-66] Consequently, it is important to consider both burnout and work engagement when addressing job functioning as a whole.

Finally, our study has some limitations. Firstly, although theory and research point to causal relationships between demands and burnout and resources and work engagement,[12,67] our design does not allow to draw causal conclusions. It is possible that the proposed relationships in the JD-R model are reversed. For instance, feelings of exhaustion and cynicism may change the way employees perceive their work demands, intensifying the feeling that demands are piling up.[68-69] Future research could use multi-wave designs that can provide insight into the development of study variables over time and the causal dynamics in this process[70]. Secondly, all data have been gathered using self-report questionnaires. This might lead to a so-called 'common-method bias'.[71] A potential way to reduce this bias would be to expand the sources of information (e.g., supervisors' assessments of employee burnout and engagement) and the methods of data collection (i.e., triangulation of data), for instance by including qualitative data as a next step. We are aware that self- and observer ratings of

For peer review only - http://bmjopen.bmj.com/site/about/guidelines.xhtml

work characteristics and job demands may not necessarily correlate high[12]. However, we believe that expanding the information source through third-party observations as well as triangulation can help to provide a richer picture of the work characteristics being studied. Another limitation of this study is the relatively small sample size, especially in the group of specialists. To examine if power was sufficient in both samples of residents and specialists, we conducted post-hoc power analyses[e] on several unsupported direct effects (e.g., paths self-compassion and psychological flexibility to burnout for specialists, path psychological capital to burnout for residents). In all cases, the statistical power was 1.0, indicating that non-significant findings are most likely truly non-significant, i.e., that this study had enough power for the conducted analyses. Furthermore, this study is limited by its sample composition, which predominantly consists of female pediatricians or pediatric residents. While the gender demographics of our sample closely matched the broader hospital population for residents, this was not the case for specialists as female specialists were overrepresented in our sample. While this is likely due to the intervention context of this study, future studies should include a larger sample with different specialties and ensure a more equal gender distribution to test if these effects are stable across specialties and gender. However, with ample evidence supporting the JD-R model's presumptions in different professional contexts and in various samples, it is not likely that the results of this study are greatly biased by its sample characteristics. Finally, the concept of colleague support is limited in the sense that it does not allow for a differentiation between support functions. Ideally, a concept of social support including such a differentiation[63] could help to disentangle *how* perceived social support helps specialists to counteract stress and exhaustion and promote work engagement.

[e] Soper DS. (2019). Post-hoc Statistical Power Calculator for Multiple Regression [Software]. Available from http://www.danielsoper.com/statcalc

For peer review only - http://bmjopen.bmj.com/site/about/guidelines.xhtml

Implications

To our knowledge, this is the first study that attempts to reveal the specific demands and resources that may impact burnout and work engagement among residents and specialists. Understanding how demands and resources are linked to physician well-being and engagement is a fundamental premise for designing successful interventions to minimize the risk of burnout. Our results suggest that a one-size-fits-all approach might not be effective for promoting physician well-being but, instead, that interventions should be tailored to the specific needs of specialists and residents.[2] This is in line with a recent call to consider contextual complexities such as specialty or career stage when setting up interventions to promote physician well-being.[19] While interventions for specialists should focus on increasing psychological capital and colleague support, interventions for residents should, in addition to increasing psychological capital, be aimed at increasing, self-compassion and psychological flexibility. Interestingly, especially personal resources seemed to preserve physician well-being and engagement. Therefore, targeting personal resources rather than structural constraints seems promising to counter the demands physicians face. Additionally, interventions could also target training institutions and hospitals with the aim of building a culture that facilitates self-compassion, psychological capital, and psychological flexibility among their residents and specialists. We consider testing the effectiveness of interventions aiming at fostering personal resources an important future inquiry.

CONCLUSION

With physician well-being being central to optimal patient care, it is important to uncover work characteristics that influence work engagement and burnout. This study revealed that physicians are not a uniform body, but that medical residents and specialists face different challenges in their work that require unique resources to resort to. While all physicians are

For peer review only - http://bmjopen.bmj.com/site/about/guidelines.xhtml

likely to benefit from resources facilitating goal attainment (i.e., psychological capital), medical residents may additionally benefit from self-care and flexibility, and specialists may additionally benefit from social support. Finally, by respecting also the unique needs of residents and specialists, one can create equal opportunities for all physicians in the challenging workplace that healthcare is.

For peer review only

For peer review only - http://bmjopen.bmj.com/site/about/guidelines.xhtml

Acknowledgments

The authors wish to thank all the residents and attending physicians who participated in this study.

Contributors

L Solms, A E M van Vianen, T Theeboom, J Koen, A P J de Pagter, M de Hoog contributed to the design and conception of the study. All authors were involved in the implementation of the study and the acquisition of the data. L Solms, A E M van Vianen, J Koen analyzed the data, and interpreted the data together with T Theeboom, A P J de Pagter, and M de Hoog. All authors reviewed and approved the manuscript.

Funding/Support

This research received no specific grant from any funding agency in the public, commercial or not-for-profit sectors.

Competing interests

None declared.

Ethical approval

The institutional Ethic Review Board of the University of Amsterdam waived ethical approval for this study, on December 12, 2016; document 2016-WOP-7521.

Disclaimer

None reported.

Data sharing statement

Data and statistical code are available upon request. Any queries should be directed to the first author.

Previous presentations

This research has been presented at the annual national conference of the Netherlands Association for Medical Education (NVMO) in Egmond aan Zee, The Netherlands (November 16, 2018).

For peer review only

REFERENCES

1. Schaufeli WB, Salanova M, González-Romá V, Bakker AB. The measurement of engagement and burnout: A two sample confirmatory factor analytic approach. J Happiness Stud. 2002; 3:71-92.
2. Scheepers RA, Lases LS, Arah OA, Heineman MJ, Lombarts KM. Job resources, physician work engagement, and patient care experience in an academic medical setting. Acad Med. 2017; 92:1472-1479.
3. Thomas NK. Resident burnout. JAMA. 2004; 292:2880-2889.
4. Firth-Cozens J. Doctors, their wellbeing, and their stress: It's time to be proactive about stress - and prevent it. BMJ. 2003; 326:670.
5. Maslach C, Jackson SE. The measurement of experienced burnout. J Organ Behav. 1981; 2:99-113.
6. Shanafelt TD, Hasan O, Dyrbye L, et al. Changes in burnout and satisfaction with work-life balance in physicians and the general US working population between 2011 and 2014. Mazo Clin Proc. 2015; 90:1600-1613.
7. Dyrbye LN, Varkey P, Boone SL, Satele DV, Sloan JA, Shanafelt TD. Physician satisfaction and burnout at different career stages. Mayo Clin Proc. 2013; 88:1358-1367.
8. Prins JT, Hoekstra-Weebers JEHM, Van De Wiel HBM, et al. Burnout among Dutch medical residents. Int J Behav Med. 2007; 14:119-125.
9. Shanafelt TD, Bradley KA, Wipf JE, Back AL. Burnout and self-reported patient care in an internal medicine residency program. Ann Intern Med. 2002; 136:358-367.
10. Rimmer A. Employers must tackle high level of burnout among trainees, says GMC. BMJ. 2018; 362:k3018.
11. Campbell J, Prochazka AV, Yamashita T, Gopal R. Predictors of persistent burnout in internal medicine residents: a prospective cohort study. Acad Med. 2010; 85:1630-1634.

For peer review only

For peer review only - http://bmjopen.bmj.com/site/about/guidelines.xhtml

12. Demerouti E, Bakker AB, Nachreiner F, Schaufeli WB. The job demands-resources model of burnout. J Appl Psychol. 2001; 86:499-512.

13. Christian MS, Garza AS, Slaughter JE. Work engagement: A quantitative review and test of its relations with task and contextual performance. Pers Psychol. 2011; 64:89-136.

14. Salanova M, Agut S, Peiró JM. Linking organizational resources and work engagement to employee performance and customer loyalty: the mediation of service climate. J Appl Psychol. 2005; 90:1217-1227.

15. Loerbroks A, Glaser J, Vu-Eickmann P, Angerer P. Physician burnout, work engagement and the quality of patient care. Occup Med. 2017; 67:356-362.

16. Prins JT, Van Der Heijden FMMA, Hoekstra-Weebers JEHM, et al. Burnout, engagement and resident physicians' self-reported errors. Psychol Health Med. 2009; 14:654-666.

17. Maslach C, Leiter MP. The truth about burnout: how organizations cause personal stress and what to do about it. San Francisco. CA: Jossey-Bass; 1997

18. Schaufeli WB, Bakker AB. Job demands, job resources, and their relationship with burnout and engagement: A multi-sample study. J Org Behav. 2004; 25:293-315.

19. Carrieri D, Briscoe S, Jackson M, et al. 'Care Under Pressure': a realist review of interventions to tackle doctors' mental ill-health and its impacts on the clinical workforce and patient care. BMJ Open, 2018; 8:e021273.

20. Bakker AB, Demerouti E. The job demands-resources model: State of the art. J Manage Psychol. 2007; 22:309-328.

21. Xanthopoulou D, Bakker AB, Demerouti E, Schaufeli WB. Reciprocal relationships between job resources, personal resources, and work engagement. J Vocat Behav. 2009; 74:235-244.

22. Richter P, Hacker W. Workload and strain: Stress, fatigue, and burnout in working life. Heidelberg, Germany: Asagner; 1998.

For peer review only

For peer review only - http://bmjopen.bmj.com/site/about/guidelines.xhtml

23. Bakker AB, Demerouti E, Euwema MC. Job resources buffer the impact of job demands on burnout. J Occup Health Psych. 2005; 10:170-180.

24. Hobfoll SE. Conservation of resources: A new attempt at conceptualizing stress. Am Psychol. 1989*;* 44:513-524.

25. Hobfoll SE, Johnson RJ, Ennis N, Jackson AP. Resource loss, resource gain, and emotional outcomes among inner city women. J Pers Soc Psychol. 2003; 84:632-643.

26. Xanthopoulou D, Bakker AB, Demerouti E, Schaufeli WB. The role of personal resources in the job demands-resources model. Int J Stress Manag. 2007; 14:121-141.

27. Hakanen JJ, Bakker AB, Schaufeli WB. Burnout and work engagement among teachers. J Sch Psychol. 2006; 43:495-513.

28. Halbesleben JR. A meta-analysis of work engagement: Relationships with burnout, demands, resources, and consequences. Work engagement: A handbook of essential theory and research. 2010; 8:102-117.

29. Murray MA, Cardwell C, Donnelly M. GPs' mental wellbeing and psychological resources: a cross-sectional survey. Br J Gen Pract. 2017; 67:e547-e554

30. Van den Heuvel M, Demerouti E, Bakker AB, Schaufeli WB. Personal resources and work engagement in the face of change. In: Houdmont J, Leka S. Contemporary occupational health psychology: Global perspectives on research and practice. Hoboken, New Jersey: John Wiley & Sons; 2010:124-150.

31. Crawford ER, LePine JA, Rich BL. Linking job demands and resources to employee engagement and burnout: a theoretical extension and meta-analytic test. J Appl Psychol. 2010; 95:834-848.

32. Spector PE, Jex SM. Development of four self-report measures of job stressors and strain: interpersonal conflict at work scale, organizational constraints scale, quantitative

For peer review only - http://bmjopen.bmj.com/site/about/guidelines.xhtml

workload inventory, and physical symptoms inventory. J Occup Health Psychol, 1998; 3:356-367.

33. De Witte H. Job insecurity: Review of the international literature on definitions, prevalence, antecedents and consequences. SA J Ind Psychol. 2005; 31:1-6.

34. De Witte H. Arbeidsethos en jobonzekerheid: Meting en gevolgen voor welzijn, tevredenheid en inzet op het werk [Work ethics and job insecurity: Measurement and consequences for well-being, satisfaction, and work engagement]. In: Bouwen R, De Witte K, De Witte H, Taillieu T. Van groep naar gemeenschap. Liber Amicorum Prof. Dr. Leo Lagrou. Leuven, Belgium: Garant. 2000:325-350.

35. Netemeyer RG, Boles JS, McMurrian R. Development and validation of work–family conflict and family–work conflict scales. J Appl Psychol. 1996; 81:400-410.

36. Morgeson FP, Humphrey SE. The Work Design Questionnaire (WDQ): developing and validating a comprehensive measure for assessing job design and the nature of work. J Appl Psychol. 2006; 91:1321-1329.

37. Vinokur A, Schul Y, Caplan RD. Determinants of perceived social support: Interpersonal transactions, personal outlook, and transient affective states. J Pers Soc Psychol. 1987; 53:1137-1145.

38. Ouweneel E, Le Blanc PM, Schaufeli WB. Don't leave your heart at home: Gain cycles of positive emotions, resources, and engagement at work. Career Dev Int. 2012; 17:537-556.

39. Luthans F, Youssef CM, Avolio BJ. Psychological capital: Developing the human competitive edge. Oxford: Oxford University Press; 2007.

40. Neff KD, Vonk R. Self-compassion versus global self-esteem: Two different ways of relating to oneself. J Pers. 2009; 77:23-50.

41. Hayes SC, Luoma JB, Bond FW, Masuda A, Lillis J. Acceptance and commitment therapy: Model, processes and outcomes. Behav Res Ther. 2006; 44:1-25.

For peer review only

For peer review only - http://bmjopen.bmj.com/site/about/guidelines.xhtml

42. Bond FW, Lloyd J, Guenole N. The work-related acceptance and action questionnaire: Initial psychometric findings and their implications for measuring psychological flexibility in specific contexts. J Occup Organ Psychol. 2013; 86:331-347.

43. Schaufeli WB, van Dierendonck D. UBOS Utrechtse Burnout Schaal: Handleiding. Lisse, The Netherlands: Swets Test Publishers; 2000.

44. Maslach C, Jackson SE, Leiter MP, Schaufeli WB, Schwab RL. Maslach burnout inventory. Palo Alto, CA: Consulting Psychologists Press; 1986; 21:3463-3464.

45. Schaufeli WB, Buunk BP. Burnout: An overview of 25 years of research and theorizing. In: Schabracq MJ, Winnubst JAM, Cooper CL. The Handbook of Work and Health Psychology. Hoboken, New Jersyey: John Wiley & Sons; 2003; 2:383-426.

46. Schaufeli WB, Martinez IM, Pinto AM, Salanova M, Bakker AB. Burnout and engagement in university students: A cross-national study. J Cross Cult Psychol. 2002; 33:464-481.

47. Maslach C, Schaufeli WB, Leiter MP. Job burnout. Annu Rev Psychol. 2001; 52:397-422.

48. Jöreskog KG, Sörbom D. LISREL 8: Structural equation modeling with the SIMPLIS command language. Chicago, IL, US: Scientific Software International; Hillsdale, NJ, US: Lawrence Erlbaum Associates, Inc.; 1993.

49. Kline RB. Convergence of structural equation modeling and multilevel modeling. In: Williams M, Vogt WP. The SAGE handbook of innovation in social research methods. London: SAGE Publications Ltd; 2011:562-589.

50. Dyrbye LN, West CP, Satele D, et al. Burnout among US medical students, residents, and early career physicians relative to the general US population. Acad Med. 2014; 89:443-451.

For peer review only

For peer review only - http://bmjopen.bmj.com/site/about/guidelines.xhtml

51. Xanthopoulou D, Baker AB, Heuven E, Demerouti E, Schaufeli WB. Working in the sky: A diary study on work engagement among flight attendants. J Occup Health Psych. 2008; 13:345-356.

52. Lyubomirsky S, King L, Diener E. The benefits of frequent positive affect: Does happiness lead to success?. Psychol Bull. 2005; 131:803-855.

53. Avey JB, Reichard RJ, Luthans F, Mhatre KH. Meta-analysis of the impact of positive psychological capital on employee attitudes, behaviors, and performance. Hum Resour Dev Q. 2011; 22:127-152.

54. Avey JB, Luthans F, Jensen SM. Psychological capital: A positive resource for combating employee stress and turnover. Hum Resour Manage. 2009; 48:677-693.

55. Tims M, Bakker AB, Derks D. The impact of job crafting on job demands, job resources, and well-being. J Occup Health Psych. 2013; 18:230-240.

56. LePine JA, Podsakoff NP, LePine MA. A meta-analytic test of the challenge stressor-hindrance stressor framework: An explanation for inconsistent relationships among stressors and performance. Acad Manage J, 2005; 48:764-775.

57. Schaufeli WB, Bakker AB, Van der Heijden FM, Prins JT. Workaholism, burnout and well-being among junior doctors: The mediating role of role conflict. Work Stress. 2009; 23:155-172.

58. Conrad P. Learning to doctor: Reflections on recent accounts of the medical school years. J Health Soc Behav. 1988; 29:323-332.

59. Kashdan TB, Rottenberg J. Psychological flexibility as a fundamental aspect of health. Clin Psychol Rev. 2010; 30:865-878.

60. Cohen S. Social relationships and health. Am Psychol. 2004; 59:676-684.

61. Berkman LF, Glass T, Brissette I, Seeman TE. From social integration to health: Durkheim in the new millennium. Soc Sci Med. 2000; 51:843-857.

For peer review only - http://bmjopen.bmj.com/site/about/guidelines.xhtml

62. Van der Doef M, Maes S. The job demand-control (-support) model and psychological well-being: a review of 20 years of empirical research. Work Stress. 1999; 13:87-114.

63. Schaefer C, Coyne JC, Lazarus RS. The health-related functions of social support. J Behav Med. 1981; 4:381-406.

64. Zuckerman M. Attribution of success and failure revisited, or: The motivational bias is alive and well in attribution theory. J Pers. 1979; 47:245-287.

65. Schaufeli WB, Taris TW, Van Rhenen W. Workaholism, burnout, and work engagement: Three of a kind or three different kinds of employee well-being?. Appl Psychol-Int Rev. 2008; 57:173-203.

66. Høigaard R, Giske R, Sundsli K. Newly qualified teachers' work engagement and teacher efficacy influences on job satisfaction, burnout, and the intention to quit. Eur J Teach Educ. 2012; 35:347-357.

67. Montgomery AJ, Panagopolou E, Benos A. Work–family interference as a mediator between job demands and job burnout among doctors. Stress Health. 2006; 22:203-212.

68. Leiter MP, Durup MJ. Work, home, and in-between: A longitudinal study of spillover. J Appl Behav Sci. 1996; 32:29-47.

69. Demerouti E, Bakker AB, Bulters AJ. The loss spiral of work pressure, work–home interference and exhaustion: Reciprocal relations in a three-wave study. J Vocat Behav. 2004; 64:131-149.

70. De Lange, A. H. (2005). What about causality?: examining longitudinal relations between work characteristics and mental health. Unpublished dissertation. Nijmegen, The Netherlands. Radboud University Press; 2005.

71. Podsakoff PM, MacKenzie SB, Lee JY, Podsakoff NP. Common method biases in behavioral research: A critical review of the literature and recommended remedies. J Appl Psychol. 2003; 88:879-903.

For peer review only - http://bmjopen.bmj.com/site/about/guidelines.xhtml

Figure Legends

Figure 1

Tested path model of job demands (workload, job insecurity, work-life conflict), job resources (autonomy, colleague support, supervisor support), personal resources (psychological capital, self-compassion, psychological flexibility), tenure, burnout, and work engagement in a Study on the Role of Personal Resources for Work Engagement and Burnout, 2017[a-f]

For peer review only

For peer review only - http://bmjopen.bmj.com/site/about/guidelines.xhtml

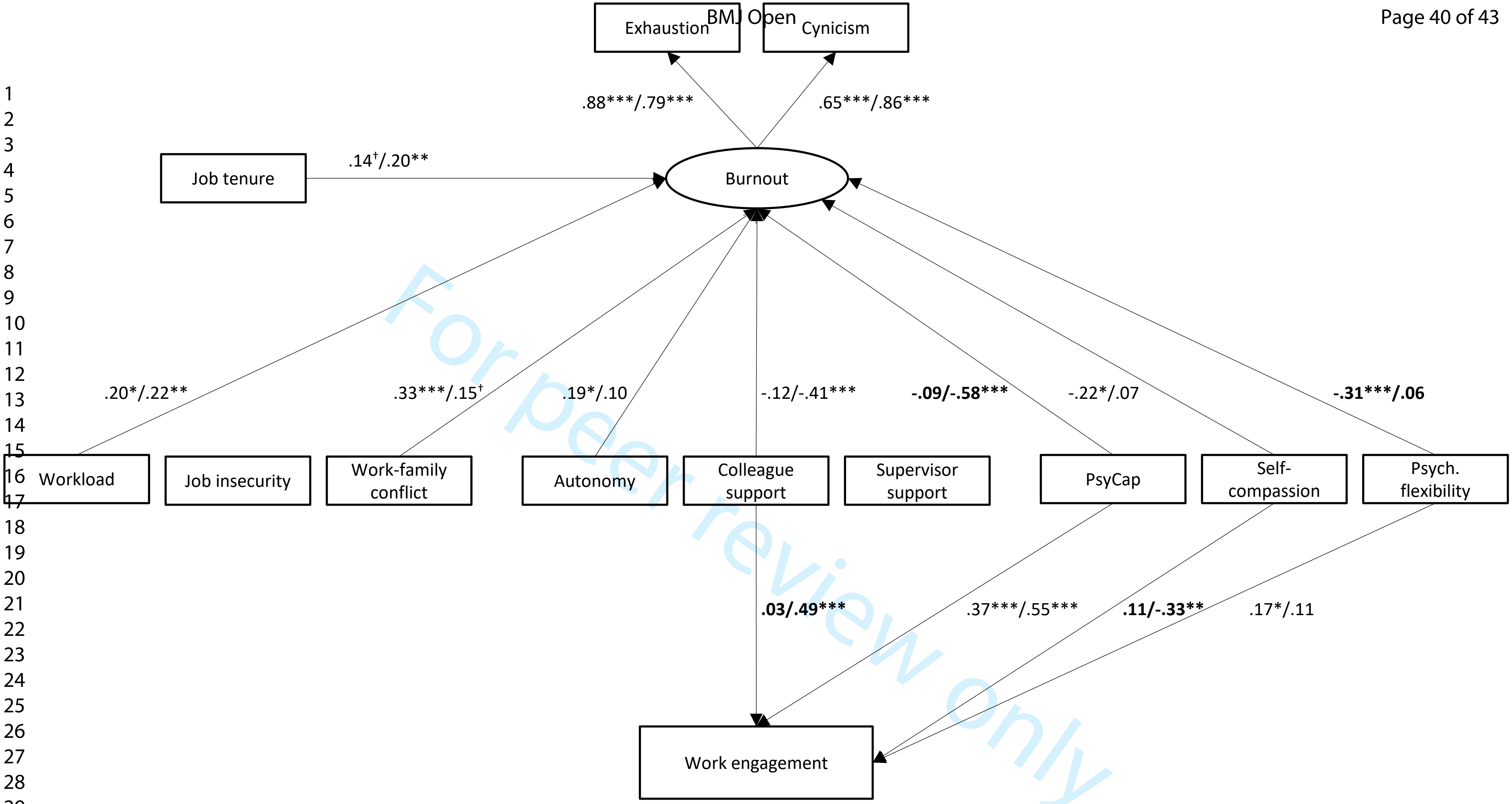

Abbreviation: PsyCap indicates psychological capital; psych. flexibility indicates psychological flexibility; the direction of the paths is indicated by an arrow symbol.
[a]Burnout as latent variable is based on the observed variables exhaustion and cynicism.
[b]The authors allowed covariation between all predictor variables, as well as between job tenure and autonomy. They also allowed covariation of error variance between exhaustion and cynicism with work engagement. For ease of reading, covariation is not depicted in the model.
[c]Only significant paths are displayed; the paths from job insecurity and supervisor support to burnout were not significant. Also, the paths from work-family conflict, autonomy, supervisor support, job insecurity and workload to work engagement were not significant.
[d]Standardized parameter estimates refer to residents (first) and specialists (second).
[e]Statistical significance of parameter estimates is indicated by the following symbols: $^{\dagger}p < .10$, $^{*}p < .05$, $^{**}p < .01$, $^{***}p < .001$.
[f]Bold parameter estimates depict significant differences in model estimates between residents and specialists.

Reporting checklist for cross sectional study.

Based on the STROBE cross sectional guidelines.

Instructions to authors

Complete this checklist by entering the page numbers from your manuscript where readers will find each of the items listed below.

Your article may not currently address all the items on the checklist. Please modify your text to include the missing information. If you are certain that an item does not apply, please write "n/a" and provide a short explanation.

Upload your completed checklist as an extra file when you submit to a journal.

In your methods section, say that you used the STROBE cross sectional reporting guidelines, and cite them as:

von Elm E, Altman DG, Egger M, Pocock SJ, Gotzsche PC, Vandenbroucke JP. The Strengthening the Reporting of Observational Studies in Epidemiology (STROBE) Statement: guidelines for reporting observational studies.

		Reporting Item	Page Number
Title	#1a	Indicate the study’s design with a commonly used term in the title or the abstract	1
Abstract	#1b	Provide in the abstract an informative and balanced summary of what was done and what was found	2-3
Background / rationale	#2	Explain the scientific background and rationale for the investigation being reported	4-6
Objectives	#3	State specific objectives, including any prespecified hypotheses	7
Study design	#4	Present key elements of study design early in the paper	7-8
Setting	#5	Describe the setting, locations, and relevant dates, including periods of recruitment, exposure, follow-up, and data collection	7
Eligibility criteria	#6a	Give the eligibility criteria, and the sources and methods of selection of participants.	7
	#7	Clearly define all outcomes, exposures, predictors, potential confounders, and effect modifiers. Give diagnostic criteria, if applicable	8-11
Data sources / measurement	#8	For each variable of interest give sources of data and details of methods of assessment (measurement). Describe comparability of assessment methods if there is more than one group. Give information separately for for exposed and unexposed groups if applicable.	7

For peer review only

Bias	#9	Describe any efforts to address potential sources of bias	25
Study size	#10	Explain how the study size was arrived at	7
Quantitative variables	#11	Explain how quantitative variables were handled in the analyses. If applicable, describe which groupings were chosen, and why	11
Statistical methods	#12a	Describe all statistical methods, including those used to control for confounding	11-12
	#12b	Describe any methods used to examine subgroups and interactions	11
	#12c	Explain how missing data were addressed	See note 1
	#12d	If applicable, describe analytical methods taking account of sampling strategy	n/a
	#12e	Describe any sensitivity analyses	n/a
Participants	#13a	Report numbers of individuals at each stage of study—eg numbers potentially eligible, examined for eligibility, confirmed eligible, included in the study, completing follow-up, and analysed. Give information separately for for exposed and unexposed groups if applicable.	12
	#13b	Give reasons for non-participation at each stage	n/a
	#13c	Consider use of a flow diagram	n/a
Descriptive data	#14a	Give characteristics of study participants (eg demographic, clinical, social) and information on exposures and potential confounders. Give information separately for exposed and unexposed groups if applicable.	12-13
	#14b	Indicate number of participants with missing data for each variable of interest	n/a
Outcome data	#15	Report numbers of outcome events or summary measures. Give information separately for exposed and unexposed groups if applicable.	14, 16
Main results	#16a	Give unadjusted estimates and, if applicable, confounder-adjusted estimates and their precision (eg, 95% confidence interval). Make clear which confounders were adjusted for and why they were included	17-20
	#16b	Report category boundaries when continuous variables were categorized	n/a
	#16c	If relevant, consider translating estimates of relative risk into absolute risk for a meaningful time period	n/a
Other analyses	#17	Report other analyses done—e.g., analyses of subgroups and interactions, and sensitivity analyses	n/a

For peer review only

Key results	#18	Summarise key results with reference to study objectives	20-21
Limitations	#19	Discuss limitations of the study, taking into account sources of potential bias or imprecision. Discuss both direction and magnitude of any potential bias.	24-25
Interpretation	#20	Give a cautious overall interpretation considering objectives, limitations, multiplicity of analyses, results from similar studies, and other relevant evidence.	21-24
Generalisability	#21	Discuss the generalisability (external validity) of the study results	26
Funding	#22	Give the source of funding and the role of the funders for the present study and, if applicable, for the original study on which the present article is based	28

Author notes

1. 12 - incomplete cases were excluded from the analyses.

The STROBE checklist is distributed under the terms of the Creative Commons Attribution License CC-BY. This checklist was completed on 12. April 2019 using https://www.goodreports.org/, a tool made by the EQUATOR Network in collaboration with Penelope.ai

For peer review only
